# Microbial diversity and methanogenic activity of Antrim Shale formation waters from recently fractured wells

**DOI:** 10.3389/fmicb.2013.00367

**Published:** 2013-12-06

**Authors:** Cornelia Wuchter, Erin Banning, Tracy J. Mincer, Nicholas J. Drenzek, Marco J. L. Coolen

**Affiliations:** ^1^Marine Chemistry and Geochemistry Department, Woods Hole Oceanographic InstitutionWoods Hole, MA, USA; ^2^Reservoir Geosciences Department, Schlumberger Doll ResearchCambridge, MA, USA

**Keywords:** Antrim gas shale, biogenic gas, formation water, fermenting bacteria, methanogens

## Abstract

The Antrim Shale in the Michigan Basin is one of the most productive shale gas formations in the U.S., but optimal resource recovery strategies must rely on a thorough understanding of the complex biogeochemical, microbial, and physical interdependencies in this and similar systems. We used Illumina MiSeq 16S rDNA sequencing to analyze the diversity and relative abundance of prokaryotic communities present in Antrim shale formation water of three closely spaced recently fractured gas-producing wells. In addition, the well waters were incubated with a suite of fermentative and methanogenic substrates in an effort to stimulate microbial methane generation. The three wells exhibited substantial differences in their community structure that may arise from their different drilling and fracturing histories. Bacterial sequences greatly outnumbered those of archaea and shared highest similarity to previously described cultures of mesophiles and moderate halophiles within the *Firmicutes*, *Bacteroidetes*, and δ- and ε-*Proteobacteria.* The majority of archaeal sequences shared highest sequence similarity to uncultured euryarchaeotal environmental clones. Some sequences closely related to cultured methylotrophic and hydrogenotrophic methanogens were also present in the initial well water. Incubation with methanol and trimethylamine stimulated methylotrophic methanogens and resulted in the largest increase in methane production in the formation waters, while fermentation triggered by the addition of yeast extract and formate indirectly stimulated hydrogenotrophic methanogens. The addition of sterile powdered shale as a complex natural substrate stimulated the rate of methane production without affecting total methane yields. Depletion of methane indicative of anaerobic methane oxidation (AMO) was observed over the course of incubation with some substrates. This process could constitute a substantial loss of methane in the shale formation.

## Introduction

Microbial gas formation through decomposition of sedimentary organic matter (OM) comprises roughly 20% of the world's natural gas resources (Rice, 1992), and it is estimated that even more microbial gas is retained in the corresponding source rocks of unconventional biogenic gas shales (Milkov, 2011). In the U.S. unconventional gas resources account for nearly 10% of the total natural gas generation (Martini et al., 2003). The Michigan Basin, centered on the lower peninsula of Michigan in the U.S., is home of the Devonian (~380 Ma-old) Antrim Shale formation, one of the most productive gas shale formations in the U.S. The finely laminated Antrim Shale contains thermally immature OM with a total organic carbon (TOC) content of 0.5–24% (Shurr and Ridgley, 2002). In the central and eastern Michigan basin the Antrim shale contains thermogenic gas, created by pressure and thermal cracking of OM. Here, gas production rates in drilled wells are low and economically not successful (Martini et al., 2008). In contrast, shale gas production along the northern margin of the Michigan Basin is high and became a target area of rapid development starting with 100 wells in 1985 to over 12,000 gas-and water producing wells installed today (Martini et al., 2004; DEQ, 2012). Previous geochemical studies provided evidence for biologically mediated methane generation in the recent geological past along the northern margin of the Michigan Basin (Martini et al., 1996). Dilution of deep basin brines with meteoric waters and deep groundwater recharge during Pleistocene glaciations resulted in a steep salinity gradient in Antrim Shale pore waters with extremely diluted water along the margins to greater than 5 M NaCl at the center of the basin (McIntosh et al., 2002). The Antrim shale has been hydrologically isolated from surface water for at least 7000 years (Martini et al., 1998; McIntosh et al., 2002) and the formation water lacks abundant inorganic electron acceptors other than carbon dioxide, including sulfate and iron oxyhydroxides (Waldron et al., 2007). Such conditions are favorable for methanogens which often thrive in environments where carbon dioxide is the sole available electron acceptor (Whitman et al., 2006). Although the high salt concentrations in the Antrim formation water inhibit many microorganisms, halophilic organisms can thrive under a wide range of NaCl concentrations (Oren, 1999). A recent study on enrichment cultures from formation water of the methane-generating zone of the Antrim shale provided evidence of halophilic methanogenic communities growing at up to 2.5 M NaCl concentrations (Waldron et al., 2007), indicating that active methanogenesis is an ongoing process in the northern margins of the Antrim gas shale.

Although little is known of microbial communities in gas shale formations, similarly hydrocarbon-rich, anaerobic environments such as petroleum reservoirs and subsurface coal beds have been relatively well-studied (Magot et al., 2000; Strąpoć et al., 2008, 2011). Similar to the Antrim Shale, carbon dioxide is the predominantly bioavailable electron-acceptor in coal bed formation water (Strąpoć et al., 2011). Several studies have demonstrated the presence of active methanogenic archaea in coal beds (Shumkov et al., 1999; Green et al., 2008; Harris et al., 2008; Orem et al., 2010) and some have reported enhanced methane production with increases in surface area (Green et al., 2008), addition of inorganic nutrients (Harris et al., 2008) and trace elements (Ünal et al., 2012). The conversion of refractory OM to methane involves primarily the fermentation of polymers and monomers to fatty acids, organic acids, alcohols, hydrogen, and carbon dioxide. Subsequently degradation follows via secondary fermenting bacteria, homoacetogenic bacteria and acetoclastic, methylotrophic and hydrogenotrophic methanogens (Schink, 2006).

Similarly to coal bed formations, gas shale formations have shown enhanced methane production with increased surface area (Curtis, 2002). Horizontal drilling and hydraulic fracturing have now become two key technologies in shale gas exploration (Arthur et al., 2008; Kerr, 2010). During the drilling operation large volumes of drilling mud are pumped into the formation to cool and lubricate the drilling bit. Drilling mud contains cellulose, barite, and lignosulfonates (Caenn et al., 2011, www.fracfocus.org), which could serve as carbon and sulfate sources for microorganisms. Hydraulic fracturing is a widely used technique to fracture gas shale by pumping fluids and sand into production wells at high pressure (Arthur et al., 2008), resulting in enhanced methane extraction. Biocides are added to the fracturing water to control bacterial growth (Arthur et al., 2008), although recent studies have shown that bacteria can survive this treatment (e.g., Struchtemeyer and Elshahed, 2012) and additives such as polyacrylamide and sugar-based polymers might even be utilized microbially (Arthur et al., 2008). Despite the widespread utilization of the drilling and hydraulic fracturing procedures not much is known about the impact on the microbial communities present in formation water.

Here, we phylogenetically characterized the prokaryotic community in formation waters of three recently fractured gas-producing wells (denoted A3-11, B1-12, and C1-12) from the western margin (Manistee county) of the Antrim Shale to identify possible key microbial players in methanogenic shale gas production. Incubation experiments with formation water were performed to stimulate methanogenic communities using a variety of substrates. Direct methanogenic substrates tested include small organic acids, methanol, and trimethylamine (TMA). Sterile powdered shale, yeast extract, propionate, and glucose were used to test the ability of bacteria to convert these compounds into methanogenic substrates. Phylogenetic surveys of the incubation experiments were performed to identify the microbial communities that were stimulated by the various substrate additives. Methanol, TMA, and yeast extract substrates were monitored for consumption.

## Materials and methods

### Sampling and sampling location

**Figure 1:**
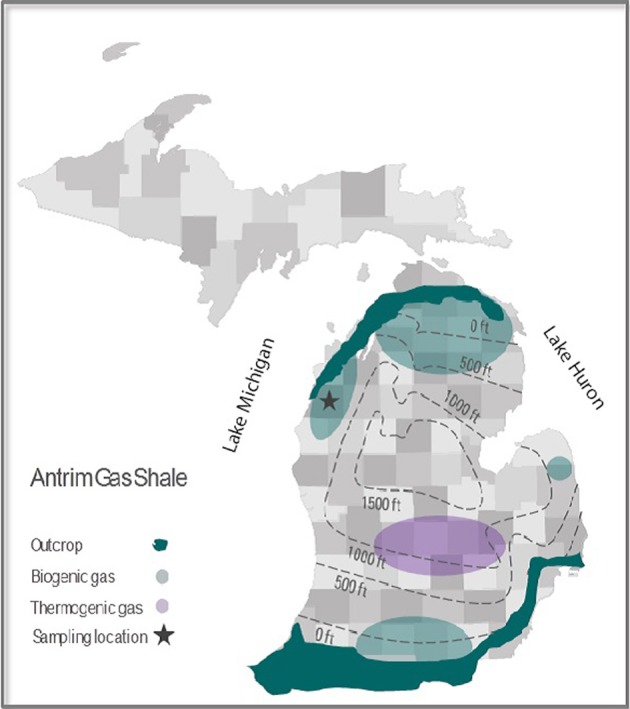
**County map of the Antrim gas shale formation with average depth contours, outcrops, and major biogenic and thermogenic production regions delineated**. The location of the wells sampled for this study is also shown.

The three investigated closely spaced (<1 km) gas-producing wells A3-11, B1-12, and C1-12 are located in the Antrim shale section of the western Michigan Basin (Manistee county) (Figure 1 and Supplementary Figure S1). All three wells were hydraulically fractured by a nitrogen foam method ~5 months prior to sampling of production water for this study. Unlike B1-12 and C1-12, A3-11 was originally drilled and brought into production more than 2 years before this study's sampling and was hydraulically fractured a total of three times, initially by different fracturing methods (Table 1). Formation water was collected at the wellhead in sterilized 1L pyrex screw cap bottles for chemical and molecular biological analyses as well as for incubation experiments. During sampling all air was removed from the collection bottles by filling them to overflowing as described previously (Huang, 2008). The anaerobic water samples were immediately placed on ice in the dark and shipped overnight to the WHOI laboratory. Well water sample bottles were transferred to an anaerobic glove box under a 95:5 N_2_:H_2_ atmosphere and inspected for accidental oxygen entrainment by adding well water subsamples to (clear and colorless) resazurin-containing media for color change. For DNA analyses biomass from 300-mL aliquots of native formation water from each well was collected on 0.2-μm Sterivex filters and kept frozen at −80°C until further analysis. Well water chemistry was measured with an ICP-MS system at the Analytical Chemical Testing Laboratory, Inc. (ACT, Mobile, AL).

**Table 1:** **Chemo-physical formation water characteristics and DNA content of the studied wells**.

**Well**	**Fracture history**	**Times fractured**	**pH**	**Alkalinity (meq.liter^−1^)**	**[Cl^−^] (M)**	**[Na^+^] (M)**	**CH_4_OH (mM)**	**[DNA] (ng L^−1^)**
A3-11	Slick water and foam fractured	3	6.97	10.6	1.72	1.26	16	1500
B1-12	Foam fractured	1	6.56	12.5	1.51	1.13	0.1	4750
C1-12	Foam fractured	1	6.69	13	1.73	1.29	4	1000

For more detailed information about the well water chemistry see Supplementary Tables S1, S2.

### Incubation experiments

The sampled anaerobic well water served as natural media for the incubation experiments. For the incubation experiments 45 mL of anaerobic well water was transferred to 140-mL sterile glass serum bottles and supplemented with 500 μL aliquots of sterilized anaerobic solutions of the appropriate substrate stock solutions. All steps involved in the set-up of the incubation experiment were performed in an anaerobic glove box (Coy Laboratory Products, Grass Lake, MI) under an atmosphere of at least 95% nitrogen and up to 5% hydrogen gas. Substrates included acetate, formate, glucose, propionate, and trimethylamine (TMA) at a final concentration of ~10 mM, yeast extract at a concentration of ~0.5 g/L, or a 0.44% (v/v) final concentration of methanol. Powdered sterile shale was also tested as a substrate to gauge the potential of the rock's native bitumen as a growth substrate, with one gram added to the appropriate incubation bottles before the addition of well water. This shale material was retrieved from a 50 m core traversing the Antrim pay zone while drilling the C1-12 well. From an organic-rich section shale material was then pulverized via mortar and pestle in the lab and autoclaved dry for addition to subsequent microbial incubations.

Incubations were conducted in duplicate for each tested substrate and monitored for up to 250 days. Each bottle also received 1 mL of a 100 mM sodium sulfide solution, 500 μL of a vitamin solution, and 100 μL of a one molar phosphate solution as additional nutrients (Whitman et al., 2006) to avoid limitation by these nutrients. Following all additions, the bottles were sealed with autoclaved butyl rubber stoppers that had been boiled for 2 h in a 2 M sodium hydroxide solution to remove any traces of methane or organic contaminants. The bottles were removed from the glove box and their headspaces purged by flowing a pressurized 80:20 nitrogen:carbon dioxide mix through 0.2-μm filters and the stoppers and then over pressured to about 100 kPa, using a bench top gassing station. All bottles were incubated in the dark at room temperature (25°C) without agitation.

A 2-mL gas-tight syringe with Luer® fitting (Valco Instruments Co. Inc., Houston, TX) was used to sample headspace gases from each incubation bottle every 1–3 weeks. For each time point, 250 μ L of headspace gas was withdrawn after 250 μ L of sterile 80:20 nitrogen:carbon dioxide mix was pushed into the bottle from the purged syringe to avoid progressive drops in gas pressure in the bottle headspaces. A similar volume replacement procedure was used for liquid samples of 1 mL, withdrawn with sterile disposable 5-mL syringes purged with the nitrogen and carbon dioxide gas mix. Every liquid sample was divided between substrate (500 μ L filtered through a 0.2-μ m filter and frozen at −20°C) and DNA (500 μ L immediately frozen at −20°C for subsequent DNA extraction).

#### Microbial gas measurements during incubations

The concentrations of methane and hydrogen in gas samples retrieved from each incubation bottle were quantified against a five-point calibration dilution of a custom 1 vol% CH_4_/H_2_ American Air Liquide standard mix on a HP 5890 Series II gas chromatograph (GC) equipped with a 2-m washed molecular sieve 13× 80/100 column and onboard thermal-conductivity and flame-ionization detectors by direct injection. Total gas abundances were then calculated from liquid and headspace volumes in each bottle and Henry's Law constants (Wilhelm et al., 1977). Anomalous readings resulting from syringe wear, identified through replicate measurements on the same or immediately preceding and succeeding sampling days, were excluded from the reported dataset. Error was propagated through these calculations using estimates of measurement error from syringes (±0.1 mL for pipetting, ±0.1 mL for 3 mL syringes, ±0.01 mL for 1 mL syringes) and standard deviations in gas concentration measurements collected from each sampling day's standard curve measurements.

#### Substrate measurements during incubations

Standard and sample dilutions for the methanol, TMA and ethanol substrates were prepared using analytical grade reagents and distilled water that had all been sparged of background volatile organic molecules (VOMs) with helium (He) for 1 h at a rate of 100 mL/min for every 1L of solution. Analyte VOMs were likewise sparged from 1:1000 diluted liquid incubation samples using a Solatek autosampler along with a Tekmar Stratum purge and trap system outfitted with a K-type trap and quantified against a 10^−5^ to 10^−8^ calibration curve of standard dilutions on an Agilent 6850 GC (Stabilwax Crossbond Carbowax PEG capillary column, 30 m × 0.25 mm ID × 0.5 μm) (Restek Chromatography Products, Bellafonte, PA) coupled to an Agilent 5975C mass spectrometer (MS) selectively monitoring ions in scan mode of *m*/*z* 32, 43, and 45 for methanol, acetone, and isopropanol, respectively. TMA measurements were performed according to manufacturer's recommendations employing an Rxi-624Sil capillary GC column (30 m × 0.25 mm ID × 1.4 μm) (Restek Chromatography Products, Bellafonte, PA). Briefly, 0.5 μl of 1000× diluted well fluid sample was injected directly into a 6850 Agilent GC, outfitted with a non-tapered injection port liner packed with deactivated glass wool (part# 5062-3587, Agilent Technologies, Santa Clara, CA) and programmed with a 12:1 split ratio. Detection of TMA was performed using an Agilent 5975C MS in scan mode monitoring the diagnostic ion of 58 amu.

Concentrations of total free amino acids (as a yeast extract proxy) and ammonium in 5×-diluted samples were measured at the Molecular Structure Facility of the University of California Davis using a Hitachi L-8800 amino acid analyzer.

### Illumina MiSeq sequencing of 16s rRNA genes: microbial diversity and relative abundance in well water and incubation experiments

DNA from the initial well water and incubation experiments were extracted according to Wuchter et al. (2004) and the total DNA was quantified fluorometrically using a Quant-iT™ PicoGreen® dsDNA Reagent (Invitrogen). The V4 region of the 16S rRNA gene was amplified with universal prokaryotic primers modified from Caporaso et al. (2012). The same reverse primer 806r was used, but in combination with the 4 bp-shorter forward primer 519f (5′-CAGCMGCCGCGGTAA-3′) (Øvreas et al., 1997), to increase the potential coverage of archaeal sequences (Wang and Qian, 2009). The theoretical coverage of the slightly modified primer combination was 88.1% of bacterial and 90.5% of archaeal 16S rDNA sequences available in the greengenes database (DeSantis et al., 2006) according to a primer coverage test using TestPrime 1.0 (Klindworth et al., 2013), as opposed to, respectively, 87.5 and 58.4% without modification of the forward primer. The formation of newly formed products was followed in real time using a Realplex quantitative PCR system (Eppendorf, Hauppauge, NY) and reagents (with the exception of primers) according to Coolen et al. (2009). The annealing temperature was set to 61°C and reactions were stopped in the exponential phase after 25–30 cycles. To minimize the formation of artifacts such as primer dimers, 10^8^ copies were subject to a second amplification reaction with the same region-specific primers that included the Illumina flowcell adapter sequences as well as the pad regions after Caporaso et al. (2012). The reverse amplification primer now also included a unique 12 base Golay barcode sequence (Caporaso et al., 2012) to support pooling of the samples. The second qPCR run was stopped after only 12 cycles when all samples reached the end of the exponential phase. The quality of the PCR products was verified by agarose gel electrophoresis and equimolar amounts of the barcoded PCR products were pooled and purified using the AMPure®XP PCR purification kit (Agencourt Bioscience Corp., Beverly, MA). Four hundred nanogram of the mixed and purified barcoded amplicons was subject to subsequent Illumina MiSeq sequencing using the facilities of Selah Genomics (Greenville, SC).

#### Quality filtering of reads, OTU picking, and taxonomic assignments

The quality scores associated with each base call for each read were used to determine the portion of each Illumina read that was of acceptable quality. Reads were first trimmed to 120 bp to avoid sequencing errors toward the end of the reads, and the minimal acceptable Phred quality score was set to 30 during demultiplexing of Fastq sequence data using the split_libraries_fastq.py script in QIIME 1.6.0 (Caporaso et al., 2010a). Furthermore, reads were discarded when they contained an N character in their sequence or barcode. The pick_otus_through_otu_table.py workflow in QIIME with default settings was used to generate an OTU table in biom format. For example, this command with default settings assigned sequences to OTUs at 97% similarity and used the RDP classifier to assign taxonomic data to each representative sequence. Then, sequences were aligned using PyNAST (Caporaso et al., 2010b) against the greengenes database and a Newick format phylogenetic tree was built for subsequent UniFrac diversity measurements. A matrix of OTU abundance in each sample with meaningful taxonomic identifiers for each OTU in biom format was assembled from the taxonomic assignments and the OTU map using default settings as the final step of the pick_otus_through_otu_table.py workflow.

#### Alpha rarefraction and beta diversity

Alpha rarefaction was performed in QIIME using the script make_rarefraction_plots.py with default parameters and using the Phylogenetic Diversity, Chao1, and observed species metrics. For beta diversity analysis we executed the jackknifed_beta_diversity.py workflow script in QIIME, which used jackknife replicates to estimate the uncertainty in principal coordinate analysis (PCoA) plots and hierarchical clustering of microbial communities. This script executed the following steps: (a) Computed a beta diversity distance matrix from the full OTU table (b), produced an Unweighted Pair Group Method with Arithmetic mean (UPGMA) tree from full distance matrix, (c) and rarefied OTU tables, (d) computed distance matrices from rarefied distance matrices, (e) compared rarefied UPGMA trees and determines jackknife support for tree nodes, (f) computed principal coordinates on each rarefied distance matrix, and (g) compared rarefied PCoA plots from each rarefied distance matrix. In this step, the jackknifed replicate PCoA plots were compared to assess the degree of variation from one replicate to the next. This variation was displayed by drawing confidence ellipsoids around the samples represented in the PCoA plots.

The diversity of bacteria and archaea in the initial well waters and incubations was furthermore analyzed using domain-specific primers targeting the entire 16S rDNA-V4 region. The resulting 465-bp-long bacterial and 396 bp-long archaeal amplicons were analyzed by denaturing gradient gel electrophoresis (DGGE) (Muyzer et al., 1993) and phylogenetic analysis of capillary sequenced (trimmed to 384 bp) individual DGGE fragments. The taxonomic information based on the longer DGGE sequences was compared with the taxonomy of the numerically most abundant OTUs from the shorter Illumina reads. Methodological details of the PCR/DGGE/capillary sequencing approach can be found in the Supplementary Info. Sequences obtained in this study were deposited to Genbank under accession numbers KC262274-KC262335 for the sequenced DGGE bands and KF72889-KF728946 for the described and discussed Illumina reads. See also supplementary information for further details.

## Results

### Well water chemistry

**Figure 2:**
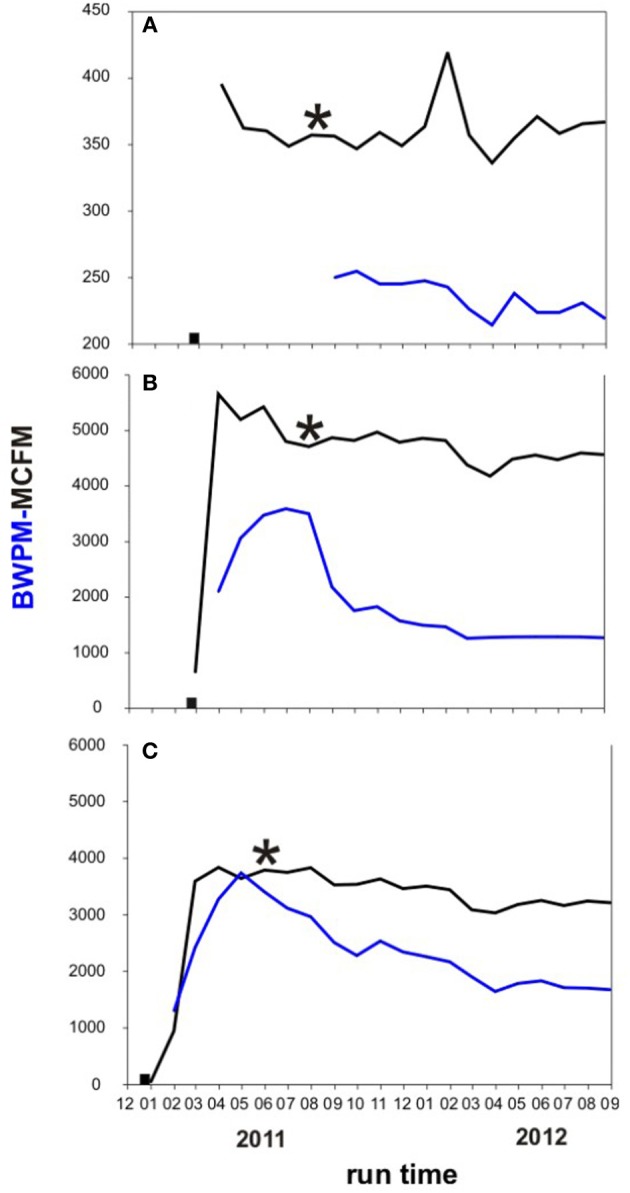
**Monthly water and gas production in the three investigated wells**. Black: Thousand cubic feet gas per month (MCFM). Blue: barrels of water per month (BWPM). Black square: time when well was fractured. Black asterisk: time when formation water was sampled for this study. **(A)** A3-11 well; **(B)** B1-12 well; **(C)** C1-12 well.

The extant water chemistry varied slightly between the wells (Table 1). In all three wells sulfate, nitrate, and phosphate were below the detection limit (<0.10 mM). Salinities ranged between 8 and 10%. Methanol concentrations differed substantially between the three wells. The highest methanol concentration was measured in the A3-11 well water (16 mM), while methanol concentrations were four times lower in C1-12 (4 mM), and 160 times lower in B1-12 well water (~0.1 mM). See Supplementary Tables S1, S2 in the supplementary info for detailed information about the analytical well water chemistry and gas composition. It is important to note that all three wells were likely sampled during a period of large flowback water production following hydraulic fracturing several months beforehand (see Figure 2; water data from A3-11 are not available for first 6 months of production). Gas and water production profiles from B1-12 and C1-12 were similar, whereas those for A3-11 were an order of magnitude smaller. Unless there were much lower formation water saturations in the A3-11 area that proportionally undermined biogenic gas generation therein, such a decline in both fluid profiles relative to the two other wells insinuates a poorer completion (i.e., less well-reservoir contact creation) in A3-11. Lack of formation water dilution may in turn explain the elevated methanol concentration measured in the waters recovered from that well.

### Prokaryotic communities in initial well waters

**Figure 3:**
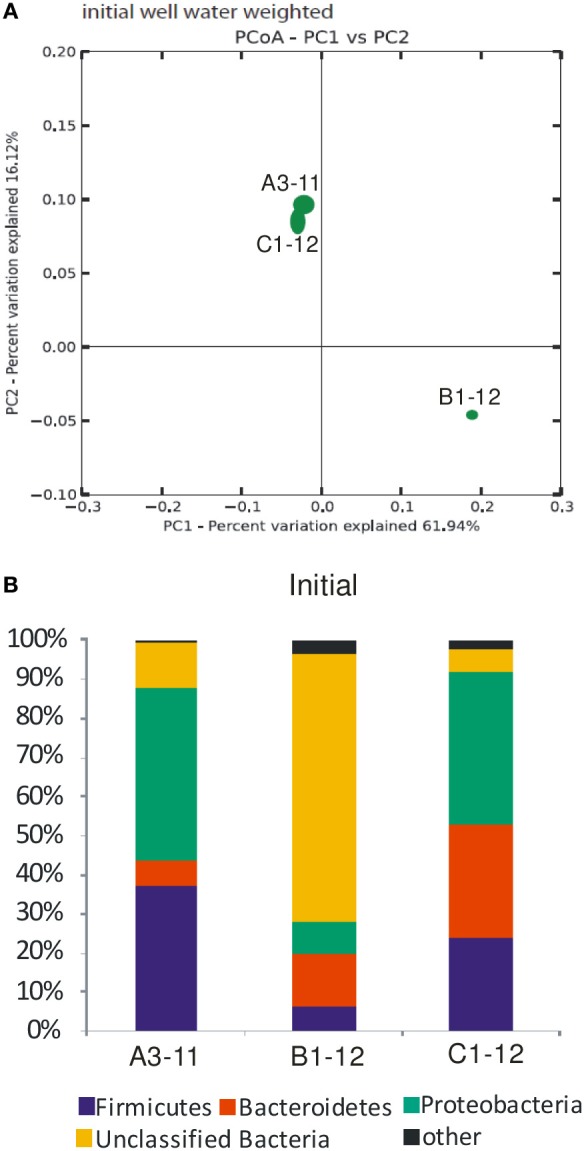
**General overview of the prokaryotic diversity in the initial well waters. (A)** Jackknifed PCoA plot of initial well water samples with weighted Unifrac. Shown is a plot of the first two principal coordinate axes, which combined explain 78% of the variation. Ellipses represent the interquartile range of the distribution of points among the 10 jackknifed replicates. **(B)** Relative abundance (% of Illumina reads) of the major bacterial phyla. A more detailed overview of the most abundant unclassified bacteria is shown in Supplementary Figure S3. “Other”; euryarchaota which comprised up to 1.3% of total Illumina reads with the remainder being less abundant bacteria (mainly OP9 and Spirochaeta).

The DNA concentration was highest (4750 ng L^−1^) in B1-12 well water, followed by 1500 ng L^−1^ in A3-11, and lowest (1000 ng L^−1^) in the C1-12 well water (Table 1) and extracted DNA served as template for molecular analyses. On average 14,533 ± 6087 high quality Illumina reads with a quality score cut-off of 30 were recovered from the initial well waters. Using a 97% sequence similarity cut-off, the total number of OTUs was twice as high in C1-12 (336 OTUs) as compared to initial well waters of A3-11 (163 OTUs) and B1-12 (164 OTUs). Rarefraction α diversity measures in the initial well waters using the phylogenetic diversity metric showed that the rarefraction curves almost reached a plateau, indicating that it becomes less likely to identify new phylotypes with greater sequencing depth (Supplementary Figure S2, Supplementary Table S3). PCoA jackknifing using weighted UniFrac distance metric showed that the prokaryotic diversity was most similar between A3-11 and C1-12 (Figure 3A). The first and second axes of the PCoA analysis explained 61.9 and 16.1%, respectively, of the prokaryotic diversity variance among the three initial well waters (Figure 3A).

#### Bacterial diversity and relative abundance

In all three wells >99% of the Illumina reads could be assigned with the RDP classifier in QIIME to the bacterial domain (Figure 3B). *Bacteroidetes*, *Proteobacteria*, *Firmicutes* and a group of unclassified bacteria comprised >96% of the Illumina reads in the studied well waters. The unclassified bacteria were most abundant in B1-12 and lowest in C1-12 (Figure 3). A single OTU with 91% similarity to the Arctic bacterium NP25 (Perreault et al., 2007) represented the majority of the unclassified bacteria, being highest (97% of the unclassified bacteria) in B1-12 (Supplementary Figure S3). This OTU represented 45% of the unclassified bacteria in C1-12, where the diversity of unclassified bacteria was highest (Supplementary Figure S3).

*Bacteroidetes*, *Proteobacteria*, and *Firmicutes*, which comprised more than 1% of the total reads shared 92–100% sequence similarity to previously described cultures of mesophilic and moderately halophilic bacterial species (Table 2). The majority of *Bacteroidetes* and *Firmicutes* showed highest sequence similarity with strictly anaerobic, fermenting bacteria, which can utilize a wide variety of organic compounds such as sugars, peptides, amino acids, organic acids, or alcohols. The dominant *Firmicutes* were most similar to described species of the order Halanaerobiales, including *Halanaerobium hydrogeniformans* (Brown et al., 2011), *Haloanaerobium congolense* (Ravot et al., 1997), *Halocella cellulolsilytica* (Simankova et al., 1993), *Orenia marismortui* (Rainey et al., 1995), *and O. salinaria* (Mouné et al., 2000) (Table 2). OTUs most similar to *Marinilabilia salmonicolor* (Nakagawa and Yamasato, 1996) and *Cytophaga* sp. AN-B14 (Daffonchio et al., 2006) comprised the most abundant *Bacteroidetes* (Table 2). The most abundant OTUs belonging to the δ- and ε-Proteobacteria were related to sulfur-, sulfate-, iron-, or nitrate-reducing bacteria (Table 2) and a single OTU with 100% sequence similarity to the nitrate-reducing *Acrobacter marinus* (Kim et al., 2010) greatly outnumbered other ε-Proteobacteria as well as the δ-Proteobacteria in all three wells (Table 2). Bacterial OTUs that represented more than 1% of the Illumina reads were also recovered via capillary sequencing of individual DGGE bands (Supplementary Table S4) and the percentage sequence similarity with the most closely related cultivated species from GenBank is shown in Supplementary Table S5. However, DGGE is at best semi-quantitative and does not provide accurate information about the relative abundance of bacterial phyla and individual OTUs in the initial well waters.

**Table 2:** **Relative abundance (% of total Illumina reads) OTUs recovered from the initial formation waters of the A3-11, B1-12, and C1-12 wells**.

**This study OTUnr**	**% of total Illumina reads**			**Phylum/class closest culture NCBI hit/accession nr**.	**Identity (%)**	**Putative function**
	**A3-11**	**B1-12**	**C1-12**			
***FIRMICUTES***						
1742	65	52.7	57.2	*Halanaerobium hydrogenoformans* [NR_074850]	99	Fermenter
437	20.9	0	15.3	*Haloanaerobium congolense* [NR_026044]	100	Fermenter, sulfur and thiosulfate reducer
1072	0	3.5	4.3	*Halocella cellulolsilytica* [NR_036959]	96	Fermenter
615	0	37.1	4.3	*Orenia marismortui* [NR_026259]	94	Fermenter
265	11.4	0	0	*Orenia salinaria* [NR_026504]	90	Fermenter
	2.7	6.7	7.2	Other		
***BACTEROIDETES***						
1364	27	90.7	70.7	*Marinilabilia salmonicolor* [AB680721]	100	Fermenter
1443	63.5	1.7	22.4	*Cytophaga* sp. AN-B14 [AM157648]	100	Fermenter
975	0	2.1	0	Bacteroidetes bacterium G13a-B [FN397996]	100	Acetogen
138	4	0	0	*Prolixibacter bellariivorans* [AB541983]	95	Fermenter
805	0	1.1	0	Bacteroidetes Phenol-4 [AF121885]	98	Phenol-degrading
667	0	0	1.1	*Bacteroides graminisolvens* [NR_041642]	100	Fermenter
	5.5	4.4	5.8	Other		
***δ-PROTEOBACTERIA***						
665	1	1.3	0	*Desulfovibrio* sp. AND 1 [AY281344]	99	Sulfate reducer
464	0	1	0	*Desulfovibrio aespoeensis* [NR_074871]	100	Sulfate reducer
1362	0	1.9	21.5	*Desufuromusa succinoxidans* [NR_029276]	98	Sulfur reducer
1363	3.4	28.5	0	*Pelobacter carbinolicus* [NR_075013]	100	Iron reducer
323	0	1.2	2.9	*Geoalkalibacter subterraneus* [EU182247]	100	Iron reducer
99	0	12.4	0	*Geobacter hephaestius* [AY737507]	98	Sulfur and iron reducer
135	0	2.2	0	*Geothermobacter* sp. HR-1 [GQ183899]	98	Iron reducer
***ε-PROTEOBACTERIA***						
148	84.7	34.1	65.1	*Acrobacter marinus* [EU512920]	100	Nitrate reducer
1407	0	7.5	6.7	*Sulfurospirillum halorespirans* [NR_028771]	92	Sulfur reducer
1339	7.2	4.6	1.2	*Sulfurospirillum carboxydovorans* [AY740528]	99	Sulfur reducer
	3.7	5.3	2.6	Other		
**UNCLASSIFIED BACTERIA**						
772	75.1	96	41.7	Arctic bacterium Np 25 [EU196331]	88-91	Unknown
1123	0	0	9.3	Unidentified bacteria [FQ677509]	85	Unknown
	24.9	4	49	Other^*^		

Other reads which represent less than 1% of the reads in the major listed phyla. Other

* (unclassified reads; see detailed information in Supplementary Figure S3).

#### Archaea diversity and relative abundance

Archaeal sequences represented only 0.15, 0.46, and 1.07% of the total Illumina reads, respectively, in A3-11, B1-12, and C1-12 and are part of the “other” in Figure 3B. All detected archaeal sequences belonged to the phylum Euryarchaeota. The majority (20) of archaeal OTUs showed 83–100% sequence similarity to uncultured environmental clones, and 91% sequence similarity or less to cultured species (see Supplementary Table S4, for details). However, Illumina sequencing also revealed two archaeal OTUs with >99% sequence similarity to the methylotrophic methanogens *Methanolobus profundi* (Mochimaru et al., 2009) and *Methanohalophilus halophilus* (Wilharm et al., 1991). Two additional archaeal OTUs were most closely related to the hydrogenotrophic methanogens *Methanocalculus halotolerans* (Ollivier et al., 1998) and *Methanoplanus limicola* (Wildgruber et al., 1982). OTUs with the same most closely related GenBank sequences were also recovered from sequenced archaeal DGGE bands spanning the entire V4 region. A phylogenetic tree of the archaeal phylotypes recovered with the DGGE approach was prepared since the majority of the archaeal sequences are only distantly related to cultivated species (Supplementary Figure S4).

### Incubation experiments

#### Total methane yields in the different treatments

**Figure 4:**
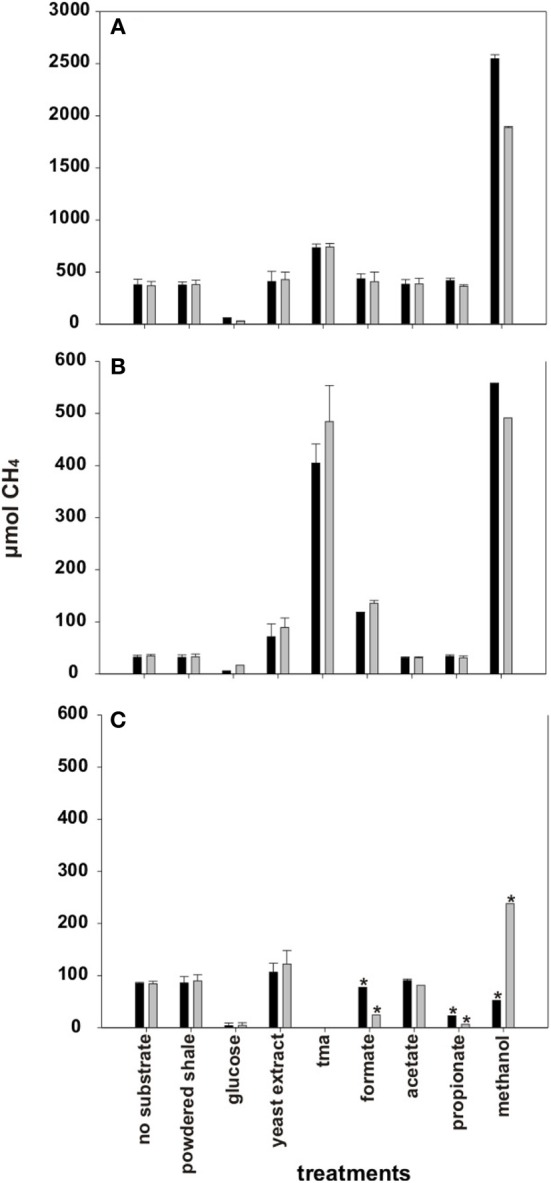
**Average methane accumulation in formation water over the course of incubation with and without added substrates. (A)** A3-11 and **(B)** B1-12 well waters were incubated for 202 days and **(C)** C1-12 well was incubated for 240 days. TMA was not tested with C1-12 well water. Note: Y-axis for **(A)** is 5X larger in magnitude than **(B,C)**. ^*^Incubation bottles where methane formation had not yet reached a plateau after 240 days of incubation. Therefore, the endpoint methane yields are shown, incubation bottle 1 (black), incubation bottle 2 (gray).

Detectable amounts of methane accumulated in the headspace of incubation bottles with all three well waters even without the addition of substrates (i.e., bottles referred to as *no substrate*) (Figure 4). The highest stable methane yields without substrate addition were measured in incubations with A3-11 well water followed by C1-12 and being lowest in B1-12. The addition of powdered shale, acetate, and propionate did not result in increased methane yields relative to incubations without substrate addition for any well (Figure 4). The addition of glucose had a negative effect on methane generation for all three wells. Formate addition stimulated methane generation only in the B1-12 well water. The addition of yeast extract had a definite stimulatory effect on methane generation in the B1-12 and C1-12 well waters. The highest methane yields were measured with TMA and methanol as substrates with five (A3-11) to ten (B1-12) fold higher methane yields in the headspace than without substrate addition (Figure 4).

#### Methanol and TMA consumption

Methanol and TMA were readily consumed by the microbial community in the incubation experiments and resulted in the highest methane yields. The utilization of methanol by methylotrophic methanogens follows according to reactions (1) and (2) as shown in Table 3. A molar methanol to methane conversion ratio [MCR_MeOH_] of 1–1.33 would be expected (Table 3) if the methanol present in incubations with or without methanol addition were utilized completely. However, the actual measured methane accumulation in the headspace was sometimes substantially lower, suggesting that not all the methanol consumed in the no-substrate and methanol incubations was converted to methane, or that methane was consumed anaerobically in some incubation bottles (Table 4).

**Table 3:** **Methanogenic consumption of methanol (reactions 1 and 2), TMA (reactions 3), and hydrogen (reaction 4) and molar conversion ratios**.

**Reaction**	**Molar conversion ratio (MCR)**
(1) CH_3_OH+H_2_ → CH_4_ + H_2_O	1.0
(2) 4 CH_3_OH → 3CH_4_ + CO_2_ + 2H_2_O	1.33
(3) 4(CH_3_) 3N + 6H_2_O →9 CH_4_ + 3 CO_2_ + 4NH_4_ +	0.45
(4) 4 H_2_ + CO_2_ → CH_4_ + 2H_2_O	4.0

**Table 4:** **Methanogenic substrate consumption and corresponding methane accumulation**.

**Treatment**	**Methanogenic substrate**	**Well**	**Bottle**	**Substrate consumed inμmol**	**Measured methane**	**MCR**
No substrate	Methanol	A3-11	1	725 (±40)	407	1.8
			2	712 (±70)	341	2.1
		C1-12	1	130 (±11)	86	1.5
			2	60 (±5)	40	1.5
Methanol	Methanol	A3-11	1	3392 (±133)	1858	1.8
			2	3174 (±130)	1361	2.3
		B1-12	1	1620 (±165)	418	3.9
			2	4140 (±240)	377	11.0
TMA^*^	TMA	A3-11	1	150 (±19)	383	0.39
			2	190 (±13.5)	404	0.47
		B1-12	1	181 (±5.1)	365	0.50
			2	144 (±15.4)	409	0.35
Yeast extract^*^	Hydrogen	B1-12	1	15	39	0.38
			2	16	45	0.36
		C1-12	1	32	22	1.45
			2	34	37	0.92
Formate^*^	Hydrogen	B1-12	1	211	74	2.90
			2	311	85	3.66

* Methane yields corrected for methane derived from methylotrophic methanogens after subtraction of the “no substrate” background. See Supplementary Figures S5, S6 for comparison. No MCR_MeOH_ could be calculated for the B1-12 ”no substrate” and C1-12 “methanol” experiments. The low initial methanol concentration in the B1-12 well reached the detection limit at the time of sampling in the incubation experiment. The C1-12 well water incubated with methanol addition did not reach a methane plateau at the end of the experiments (see Figure 4). Error for the methane measurement was on average between 1.1 and 2.7 μmol and for hydrogen measurements 0.1 and 1.4 μmol in all incubations.

TMA was tested as a methanogenic substrate for incubations with A3-11 and B1-12 well waters and resulted in methane yields comparable to those from methanol additions. TMA is utilized by methanogens according to reaction (3) as shown in Table 3. Given the stoichiometry of reaction (3), complete TMA consumption would theoretically result in a TMA to methane conversion ratio [MCR_TMA_] of 0.45. The measured methane yields, when corrected for methanol-derived methane productions, are in good agreement with theoretical expectations suggesting that most consumed TMA was converted to methane. See Supplementary Figure S5 for detailed information about the methanol and TMA consumption and methane accumulation in the A3-11 well over the course of incubation.

#### Hydrogen dynamics in the different treatments

Gradual accumulation of hydrogen in excess of 200 μmoles was detected in the headspace of all three well waters when incubated with glucose and formate. Only formate addition to B1-12 well water resulted in hydrogen consumption and concomitant methane production (Table 4). No hydrogen consumption was observed in well waters incubated with glucose, and methane accumulation was minor in all three well waters over the entire course of incubation (Figures 4 and Supplementary Figure S6). A lower amount of hydrogen (15–34 μmoles) was detected in the headspace of well water incubated with yeast-extract (Table 4) (yeast extract consumption; Supplementary Figures S6, S7). Incubation with the other substrates did not result in detectable accumulation of hydrogen.

Hydrogenotrophic methanogens utilize hydrogen according to reaction (4) as shown in Table 3. According to this stoichiometry, for every mole of methane produced, four moles of hydrogen must be consumed. When corrected for background methane production (as measured from the no-substrate bottles), for example, in the C1-12 well between 22 and 37 μmoles of methane was detected in well waters incubated with yeast extract. Such a methane yield would correspond to a range of 88 to 148 μmoles of hydrogen consumption. This is far greater than the measured hydrogen accumulations (Table 4), suggesting that part of the produced hydrogen escaped detection due to quick turnover by hydrogenotrophic methanogens in the incubations.

#### Effect of surface area

**Figure 5:**
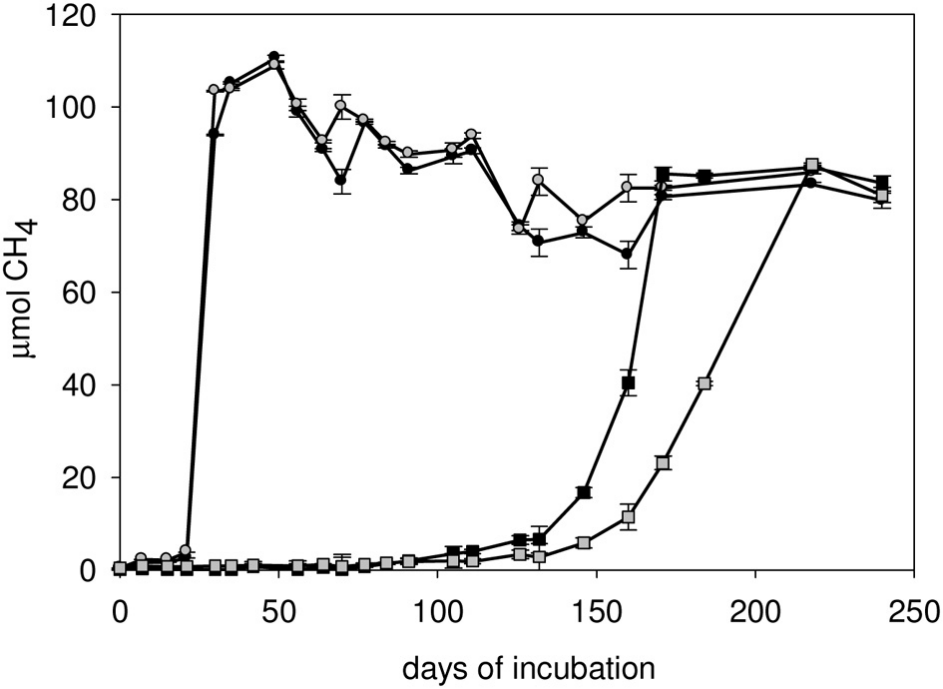
**Methane development in the C1-12 well water with sterile powdered shale as substrate (bottle 1, bottle 2), and no substrate treatment (bottle 1 and bottle 2)**.

Methane accumulated substantially earlier in incubations with powdered shale compared to well waters without substrate addition (data only shown for C1-12 well, Figure 5). Despite the difference in initial methane production rates, the amount of methane production with or without the addition of shale had stabilized at nearly identical methane yields toward the end of the incubation period. Approximately 25 μmoles of methane that was produced in C1-12 well water with sterile pulverized shale might have been consumed microbially as evident from the difference in the amount of methane after 30 and 240 days of incubation (Figure 5).

### Prokaryotic community detected in the incubation experiments

#### Bacteria

Most bacterial OTUs found in the incubation experiments were also present in the initial well water (Supplementary Figure S8). In the incubation experiments the dominant *Firmicutes* were OTUs with highest sequence similarity to *Halanerobium hydrogeniformans, H. congolense*, *Orenia marismortui, and O. salinaria*. OTUs with highest sequence similarity to *Marinilabilia salmonicolor* and *Prolixibacter bellariivorans* comprised the most abundant *Bacteroidetes*. The *Proteobacteria* were predominated by OTUs with highest sequence similarity to *Acrobacter marinus*, *Pelobacter carbinolicus*, *Sulfurospirillum* sp., and *Desulfovibrio* sp. (Supplementary Figure S8). However, the relative abundance of these microbial populations was found to differ significantly between treatments (ANOSIM; *P* = 0.024; *r* = 0.2658), which is further visualized by the limited overlap between samples in the Jacknifed PCoA plot (Supplementary Figure S9).

In all three well waters the addition of phosphate and nutrients stimulated the growth of bacteria belonging to the *Firmicutes*, reaching > 80% of the total bacterial Illumina reads (Supplementary Figure S8). Powdered shale had the largest effect on the microbial communities in C1-12 with a shift toward a predominance of *Proteobacteria* as compared to the incubations without substrate (Supplementary Figure S8). The addition of methanol stimulated *Firmicutes* in A3-11 and *Proteobacteria* in the two other well waters compared to no substrate addition. Glucose had little effect on bacterial communities in A3-11 and C-1-12, but stimulated *Proteobacteria* in B1-12. Yeast extract, TMA and formate stimulated the growth of unclassified bacteria, notably a bacterium with highest sequence similarity to the uncultured Arctic bacterium NP25, in the majority of well water incubations. Acetate and propionate stimulated the growth of *Proteobacteria* in B1-12 and C1-12, while the relative abundance of uncultured bacteria was stimulated in A3-11 upon the addition of acetate (Supplementary Figure S8).

#### Archaea

OTUs with >99% sequence similarity to the methylotrophic methanogens *Methanolobus profundi* and *Methanohalophilus halophilus*, were detected in all incubation experiments with all tested substrates. Sequences with 99% sequence similarity to the hydrogenotrophic methanogen *Methanoplanus limicola* were found in the yeast extract treatment in B1-12 and C1-12 well waters. Sequences with 100% similarity to another hydrogenotrophic methanogen, *Methanocalculus halotolerans*, were found only in formate-treated B1-12 well water. Archaeal OTUs detected in the initial well water without close cultured representatives were not detected in the incubation experiments with one exception; archaeal OTU O5 was found in methanol-treated B1-12 well water (Supplementary Table S5). Despite methane formation and the presence of OTUs related to methanogenic archaea, the relative abundance of archaeal Illumina reads did not increase in any of the incubations compared to the initial.

## Discussion

### Production water characteristics and implication on microbial community

The methane-producing zone in the Antrim Shale formation contain formation waters whose temperature and salinity range is suitable for mesophilic moderately halophilic microorganisms (Martini et al., 1996; Waldron et al., 2007). Nonetheless, as mentioned earlier, the three wells were all recently fractured and their production water profiles suggest that water samples collected for this study might have entrained volumetrically significant amounts of hydraulic fracture flowback fluid, with an especially large proportional contribution implied for the A3-11 well. Thus, the well water microbial community identified in our survey most likely represents a mixture of indigenous shale communities and allochthonous species, which were introduced to these reservoirs during drilling and fracturing procedures (Struchtemeyer et al., 2011; Struchtemeyer and Elshahed, 2012). The residence time of the introduced drilling mud and fracturing fluid into the shale formation impacts the formation water chemistry and biology. During the drilling procedure large volumes of drilling mud are often lost in the shale formation (Gray et al., 1980; Grace, 2007) and only 30–70% of fracturing fluids injected into wells are recovered in the flowback waters (Veil, 2010). A recent study on hydraulic fractured thermogenic wells in the Barnett Shale (USA) showed that the concentration of salt, iron and total dissolved solids were higher in flowback water of a well that had been in contact with fracturing fluids for 2 months compared to a well with a much shorter contact time of 24 h (Struchtemeyer and Elshahed, 2012). The authors suggested that the differences in the bacterial community in the flowback water of the two wells might be influenced by the different time intervals (2 month vs. 24 h) between fracturing and flowback at the two sites (Struchtemeyer and Elshahed, 2012). Our data support these findings. Namely, the prokaryotic beta diversity in the two wells (A3-11 and C1-12) with a longer contact time of fracturing fluid was more similar than in B1-12 with a shorter contact time of fracturing fluid as indicated by the differences in methanol concentration. In addition to methanol, cellulose, lignosulfonates, and sugar-based polymers are components of fracturing fluids and drilling muds (Caenn et al., 2011, www.fracfocus.org). For example, guar gum or hydroxyethyl cellulose is frequently used in fracturing fluids to thicken the water in order to suspend the sand. These additives might explain the presence of an active fermenting microbial community in the production water as observed in the incubation experiments by the quick utilization of fermentable substrates such as yeast extract and glucose. The prominent heterotrophs in the production water and the incubation experiments were members of the genus *Haloanaerobium* (*Firmicutes*) and the genera *Marinilabilia* and *Cytophaga* (*Bacteroidetes*). *Haloanaerobium* species usually ferment saccharides and produce H_2_, CO_2_, and C_2_ compounds (mainly acetate and sometimes ethanol, as was detected in the glucose treated incubations) (Ollivier and Cayol, 2005). One prominent OTU was closely related to *Marinilabilia salmonicolor* (Nakagawa and Yamasato, 1996), a bacterium capable of utilizing cellulose and complex carbohydrate. Additional sequences were related to *Cytophaga, Dechloromonas*, and *Pseudomonas* and members of these genera have been reported to be capable of growing on crude oil, benzene, xylene, and toluene (Prince, 2005), and might play an important role in the degradation of refractory OC in the Antrim shale. Also, the unclassified bacteria constitute a substantial fraction of the total bacterial reads, especially in the B1-12 well. These bacteria might also be capable of utilizing complex organic shale matter. Furthermore, a number of archaeal OTUs unaffiliated with known methanogenic groups were detected in the initial production water samples, which were not subsequently detected during the bottle incubations. These euryarchaeota may possess non-methanogenic metabolisms and also be involved in bitumen degradation *in situ*.

### Methanogenic capabilities in incubation experiments

Methanol was quickly utilized in the incubation experiments and high concentrations of added methanol did not inhibit methylotrophic methanogenesis suggesting that the methanogenic community is well-adapted to high methanol concentrations most likely derived from the fracturing procedure since methanol is a standard ingredient in fracturing fluids. Halophilic methanogens generally utilize substrates such as methanol, methylamines, or dimethyl sulfide, which yields more free energy compared to acetate or hydrogen (Oren, 2001). This extra energy might be used for their osmoregulatory system to balance the energetic demands of the saline environment (Oren, 2001).

TMA was below detection limit in our formation waters suggesting that this compound plays a minor role in comparison to methanol as a substrate for methylotrophic methanogens in this environment. TMA could potentially be formed in formation water through fermentation of betaine, which is an osmoregulatory compound synthesized by some halophilic bacteria (Oren, 1990). The bacterial fermentation of betaine can yield acetate and TMA. Also, similar to coal matrices (Strąpoć et al., 2011) the organic-rich Antrim Shale might be a source of methylamines.

Acetate was not utilized by methanogens in any of the three analyzed well waters and we did not find any phylogenetic evidence for acetoclastic methanogens. This is in agreement with the finding that acetoclastic and hydrogenotrophic methanogens generally thrive in freshwater or lower salinity environments (Oren, 1999, 2001). Instead, hydrogenotrophic methanogens were detected in the initial well waters and after incubation with yeast extract. Hydrogenotrophic methanogens were also identified when formate was used as a substrate, but only in B1-12. Here, hydrogen was generated by fermentation and consumed concomitant with methane production. B1-12 incubated with formate yielded an OTU closely related to *Methanocalculus halotolerans*, the most halotolerant hydrogenotrophic methanogen known to date and capable of withstanding concentrations of up to 12% NaCl (Ollivier et al., 1998). One OTU closely related to *Methanoplanus limicola* (Wildgruber et al., 1982) was detected in B1-12 and C1-12 well waters incubated with yeast extract. This methanogen tolerates salt concentrations between 0.4 and 5.4% (Wildgruber et al., 1982), which is substantially lower than the salt concentration of 8–10% in our formation waters. Most likely this OTU represents a novel hydrogenotrophic methanogen with a higher salinity tolerance. Both hydrogenotrophic methanogens might possess different hydrogen affinities since the utilization of formate resulted in a 10-fold higher hydrogen concentration then the amount of hydrogen resulting from the fermentation of yeast extract. The OTU related to *M. limicola*, might be better adapted to grow at low hydrogen concentrations, whereas the OTU related to *M. halotolerans*, is capable of thriving at elevated hydrogen concentrations. This could be similar to methanogens from rice paddies, which grow slowly if at all at high hydrogen concentrations (Sakai et al., 2007).

The low methane generation in the glucose-treated incubations could be explained by an inhibition of the methanogenic community caused by the rapid depletion of nutrients and/or the decrease in pH from the accumulation of organic acid intermediates (Strąpoć et al., 2011) as a result of bacterial fermentation. The accumulation of ethanol caused by fermentation might also have had an inhibitory effect on the methanogens present in the glucose treatment.

The increase of mineral surface area greatly stimulated methane production rates as observed from formation waters that were incubated with sterile powdered shale. However, the addition of shale did not result in consistent increases in methane yield in the C1-12 well water, and the overall methane yield in the other two well waters was identical to the no substrate background. This suggests that over the course of incubation the bitumen in the powdered shale was not utilized by fermentative prokaryotes and did not yield substrates for the methanogenic communities. In contrast, a previous enrichment experiment showed that fermentative bacteria derived from Antrim well water could be enriched by using only water-soluble shale OM and subsequent methane accumulation was detected, demonstrating that methanogenic communities can be supported by using shale derived DOM as the only source of energy and carbon (Huang, 2008). Most likely our fermenting microbial community was adapted to the presence of easy degradable carbon compounds such as those stemming from the drilling and fracturing procedure, and was not adapted to degrade bitumen from the shale.

### Methanol and methane consumption

The discrepancy between the theoretically expected methane yields based on methanol consumption and measured methane yields requires non-methanogenic methanol-utilizing metabolisms, or the ability of anaerobic oxidation of methane to be present. This could be explained by OTUs related to methanol-utilizing bacteria such as *Geoalkalibacter subterraneus* (Greene et al., 2009) present in the incubation experiments. Furthermore, some sulfate reducers are known to utilize methanol (Qatibi et al., 1991; Tarasov et al., 2011), and might be involved in methanol consumption. Also, the detected archaea without close cultured relatives, which were detected in the methanol incubation experiment, might possess the capability to utilize methanol.

The depletion of methane suggests anaerobic methane oxidation (AMO) at least in C1-12 well water incubated with powdered shale. Methane can be oxidized anaerobically by a consortium of methane-oxidizing archaea (ANME clusters), and sulfate-reducing bacteria (SRB) (Knittel and Boetius, 2009). We did find sequences of SRB in our incubation experiments, however no ANME-related sequences were recovered despite a good coverage of the universal primers used to generate template DNA for Illumina sequencing. Although the archaeal partner involved in AMO was not found, the depletion of methane in some of the incubations and possible involvement of AMO is in agreement with previous geochemical evidence for anaerobic hydrocarbon oxidation in formation water of the western margin of the Antrim shale (Martini et al., 2003). The western margin formation water contains some of the highest sulfate concentrations relative to salinity in the Antrim shale (Walter et al., 1997), and C2 and C3 gasses are enriched in δ^13^C values, suggesting that anaerobe hydrocarbon oxidation occurred in the western margin (Martini et al., 2003).

The sulfate present in the formation water is most likely derived from the shale since a recent incubation-based study showed that water-soluble organic shale material sustains SRB (Huang, 2008). This is supported by our incubation experiments where a shift toward δ- and ε-*Proteobacteria*, which comprise many iron-, sulfur-, sulfate-, and nitrate reducers, was observed with the addition of powdered shale. Sulfate can be quickly utilized by SRB, which compete for hydrogen and other low-molecular-weight substrates with methanogens. Recently, a study on formation water from wells along the northern production trend of the Antrim shale showed an increase in sulfate concentrations and SRB over the last two decades (Kirk et al., 2012). The authors explained these changes by ongoing processes driven by commercial gas production such as ground water inflow from the sulfate-rich Travers limestone, which is in contact with the Antrim shale (Kirk et al., 2012). It was furthermore suggested that this development could have negative implications for commercial gas production by creating conditions that favor growth of SRB (Kirk et al., 2012). These bacteria can compete with methanogens for substrate or, as indicated previously (Martini et al., 2003) and suggested in our study, SRB could be involved in AMO and responsible for loss of methane in the shale formation.

## Concluding remarks

Microbial community composition in closely spaced production wells can differ substantially due to local variation in both reservoir quality (e.g., lithology, matrix and natural fracture microstructure, fluid chemistry and saturation) and completion quality (e.g., hydraulic fracture design and fluid composition, well cleanup workflow, and subsequent production strategy). The microbial fermenting community in our study was capable of rapidly utilizing substrates, such as glucose and yeast extract, suggesting that they are well-adapted to decompose relatively labile OM including organic additives used in drilling and fracturing fluids. Hydrogenotrophic methanogens were detected, but the production waters from recently fractured wells appear to be dominated by methylotrophic methanogens, capable of utilizing high concentrations of methanol likely stemming from the fracturing fluids. Furthermore, we found that increased surface area stimulated methane production. However, a loss of methane over the course of incubation suggests that AMO also occurs in formation waters, which might constitute a substantial loss of methane in the shale formation.

### Conflict of interest statement

The authors declare that the research was conducted in the absence of any commercial or financial relationships that could be construed as a potential conflict of interest.
