# Supplementary material for: Microbial diversity and methanogenic activity of Antrim Shale formation waters from recently fractured wells
Source: Front Microbiol. 2013 Dec 6;4:367. doi: 10.3389/fmicb.2013.00367 (PMC3853793; doi:10.3389/fmicb.2013.00367)
Supplement: Supplementary file 1 [file Presentation1.PDF]

Supplementary information:
Microbial diversity and methanogenic activity of Antrim Shale formation waters from recently fractured wells.

Cornelia Wuchter[1*], Erin Banning[1◊], Tracy Mincer[1], Nicholas J. Drenzek[2†] and Marco J.L. Coolen[1]

[1]Woods Hole Oceanographic Institution, Marine Chemistry and Geochemistry Department, Woods Hole, MA 02543, USA. [2]Schlumberger Doll Research, Reservoir Geosciences Department, Cambridge, MA 02139, USA. [†]Now at Statoil Research Center, Unconventional Shale Oil and Gas Sector, 5020-Bergen, Norway. [◊]Now at Algenol Biofuels Inc., Bonita Springs, FL 34135, USA

Materials and methods

Bacterial and archaeal taxonomy based on capillary sequenced DGGE bands.

DNA from the initial well water and incubation experiments were extracted according to (Wuchter, *et al.*, 2004). Partial bacterial and archaeal 16S rDNA genes were amplified by polymerase chain reaction (PCR) using a Realplex quantitative PCR system (Eppendorf, Hauppauge, NY) and reagents (with the exception of primers) as described previously (Coolen, *et al.*, 2009). Bacterial 16S rDNA (V4-region) was amplified with the general primers Bac341f (Muyzer, *et al.*, 1993) and Bac806r (Takai & Horikoshi, 2000). Archaeal 16S rDNA was first amplified with the archaea-specific primers Arch 21F (DeLong, 1992) and Arch915r (Stahl & Amann, 1991), followed by a nested PCR with the universal primer 519f (complementary

reverse sequence of Parch 519r (Ovreas, *et al.*, 1997)) and the archaeal primer Arch915r (i.e., V4-region) to increase specificity. We used the SYBR®Green-based qPCR protocol after Coolen *et al.*, (2009) except for the primer annealing step, which was set to 61 °C and 40 sec for both the bacterial and archaeal primer combinations. The reactions were terminated in the exponential phase (i.e., after 23 to 30 cycles) to minimize the formation of PCR artifacts. About 10^7 gene copies served as template for a second round of PCR (only 10 cycles) using the same conditions except that the 5' end of one of the primers in each pair (Bac341f or Arch915r) included a 40-basepair long GC clamp for subsequent denaturing gradient gel electrophoresis (DGGE) (Muyzer, *et al.*, 1993) The use of the long GC clamp primers in the initial round of PCR was avoided to prevent the formation of primer dimers.

PCR-amplified bacterial and archaeal 16S rDNA products were separated by DGGE. The polyacrylamide gels (6%, wt/vol) contained a linear denaturing gradient of 20 to 70% (with 100% denaturant equaling 7 M urea and 40% formamide) for bacterial and 30 to 60% for archaeal 16S rDNA. Gels were run for 15 hours at 5 $V.cm^{-1}$ and 60 ºC using a PhorU2 system (Ingeny, Leiden, Netherlands). Afterwards, the gels were stained with SYBR®Gold (Invitrogen) and a Dark Imager (Clare Chemicals Research Inc., Dolores, CO) was used to visualize the SYBR®Gold-stained DNA (Coolen, *et al.*, 2009). Digital gel images were made using the Foto/Analyst® Express System (Fotodyne, Hartland, WI) and ImageJ software. TotalLab TL100 v2006 1D-gel analysis software (Nonlinear Dynamics, Durham, NC) was used to identify the exact vertical position of each band in order to characterize unique vs. identical bands between samples. Representative DGGE bands were then sliced from the gel with a sterile scalpel and the DNA of each gel fragment was eluted in 10 mM Tris-HCl at pH 8.0 by incubation for 24 hours at 2 ºC.

Approximately 10^7 copies of the gel-eluted 16S rDNAs were re-amplified using 18 cycles and the primer combinations listed above without the GC-clamp. These amplicons served as template for subsequent capillary sequencing reactions using the Beckman Coulter Genomics facilities (Beverly, MA). In total, 284 bacterial DGGE fragments and 225 archaeal DGGE fragments were sequenced. Bacterial forward and reverse reactions were aligned and sequencing errors removed using the Sequencher 10.7 software package. All sequences were trimmed at the same length (348 bp) using the CLC Main Workbench 6.0 software package (CLC bio, Cambridge, MA). Chimera check and identification of operational taxonomic units (OTUs) based on 97% sequence identity was performed using mothur version 1.26.0 (Schloss, *et al.*, 2009). Closely related sequences of the unique recovered bacterial OTUs were identified through a BLAST search (Altschul, *et al.*, 1990) against the NCBI-nr database.

All sequence reads obtained from DGGE fragments amplified by archaeal-specific PCR primers were manually trimmed in BioEdit v7.1.3 to the sequencing primers and merged in order to maximize the amount of sequence available for phylogenetic analysis. Low-quality sequences, as judged by the observation of large numbers of ambiguous base-calls or incoherent chromatogram peaks, were excluded from further analysis. The resulting full-length DGGE fragment sequences were imported into ARB v5.1 (Ludwig, *et al.*, 2004) using a modified version of the Silva SSU Reference database release 108 (Pruesse, *et al.*, 2007). Sequences were aligned and added by parsimony analysis to a neighbor-joining tree of full-length, high-quality archaeal 16S rRNA sequences from major euryarchaeal groups to identify clusters of phylogenetically similar sequences. Each cluster's alignment was manually curated with its most closely related sequences and the sequences exported through a mask based on the resulting alignment to mothur. The sequences were divided into clusters based on class within the

euryarchaea, and OTU analysis undertaken of each cluster separately, to maximize the number of alignable positions available. Three clusters were identified and analyzed separately for OTUs, affiliated with the Methanomicrobia, the uncultured Deep Sea Hydrothermal Vent Group (DSHVG) and other uncultured groups (including the South African Gold Mine Euryarchaeal Group). OTU analyses were conducted with cutoffs between 90 and 100% similarity, and cutoffs chosen for each group in comparison with their clustering on the tree (96% similarity cutoff for Methanomicrobia OTUs, 98% for those affiliated with the DSHVG and 96% for the other uncultured groups). Representative sequences of each archaeal OTU were added by parsimony analysis to a maximum-likelihood tree of all major euryarchaeal clades built from full-length, well-aligned sequences from the Silva reference database. Bacterial and archaeal sequences obtained from this study have been submitted to GenBank database under accession numbers KC262274-KC262335.

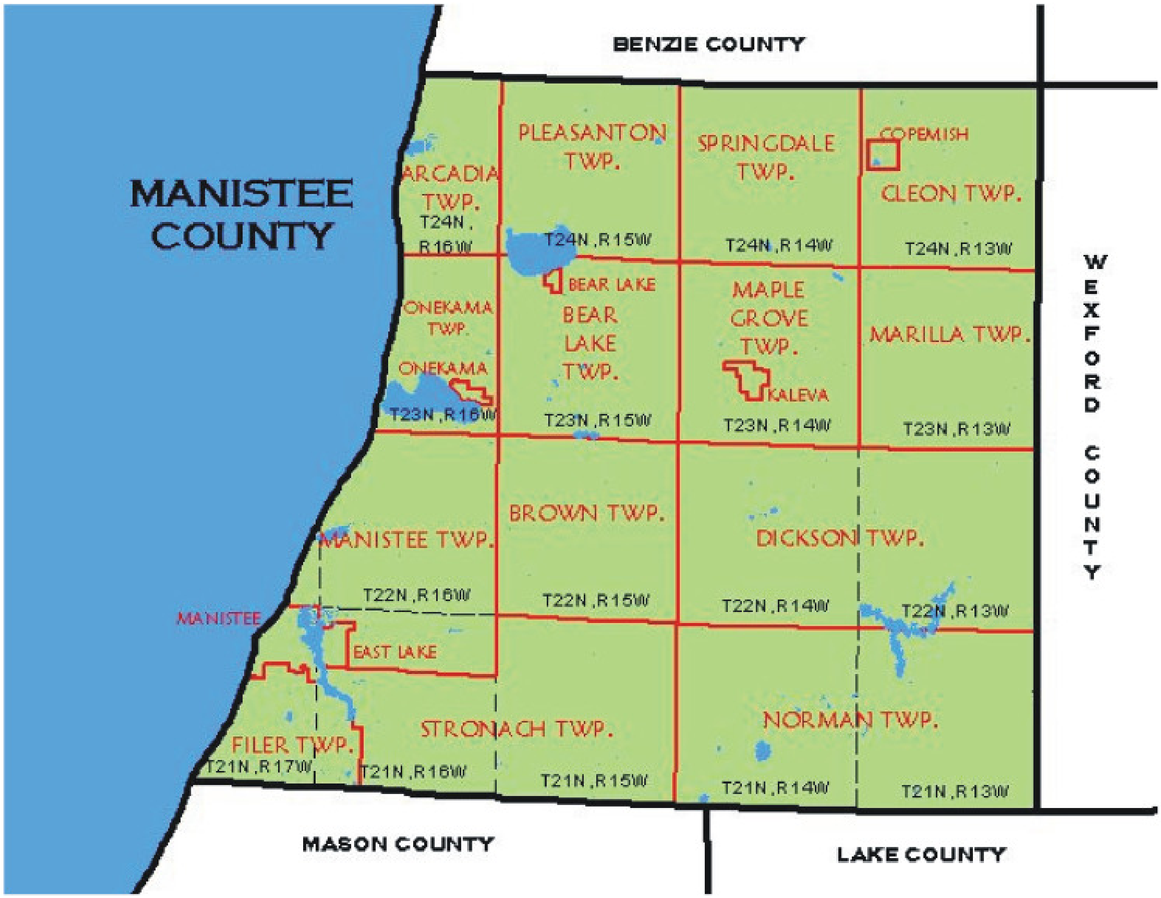

http://www.midnr.com/Publications/pdfs/ForestsLandWater/Commercial_Forest/Manistee.jpg

Supplementary Fig. S1: The three investigated wells are located in the Manistee County in the Township of Pleasanton. The wells are located in zone 11 and 12 in Pleasanton Township (well A3-11: 11-T24N-R15, well B1-12: 12-T24N-R15 and well C1-12: 12-T24N-R15). For more detailed information about a zoning map of Pleasanton see: http://www.pleasantontownship.org/zoning_files/zoningmap.gif.

Supplementary Table S1: Detailed information of the well water chemistry. Multiple fractured A3-11 well was previously analyzed in 2009 and data were kindly provided by Presidium Energy. Well water analyses for 2011 were performed for this study by ACT lab.

water sampled<br>mg/l	Presidium<br>6/29/2009<br>A3-11	ACT lab<br>7/22/2011<br>A3-11	ACT lab<br>7/22/2011<br>B1-12	ACT lab<br>5/27/2011<br>C1-12
Cations:				
Calcium	3700	3890	3160	3560
Magnesium	2300	2250	1920	2130
Sodium	26700	28900	25900	29700
Iron	51	43.2	44.1	57
Potassium	233	254	224	232
Barium	24.8	30.4	24.8	28
Strontium	160	171	150	172
Manganese	0.37	0.52	0.20	0.24
Anions:				
Bicarbonate	854	530	625	650
Sulfate	n.d	n.d	n.d	n.d
Chloride	63600	60900	53700	61200

Supplementary Table S2: Detailed gas composition of the C1-12 well was kindly provided by Presidium Energy. A3-11 and B1-12 well were not analyzed for gas composition by the company.

	mol%
Gases:	
Carbon Dioxide	3.97
nitrogen	2.916
methane	92.195
ethane	0.792
propane	0.073
iso-butane	0.052
hexane	0.001
hexane plus	0.001

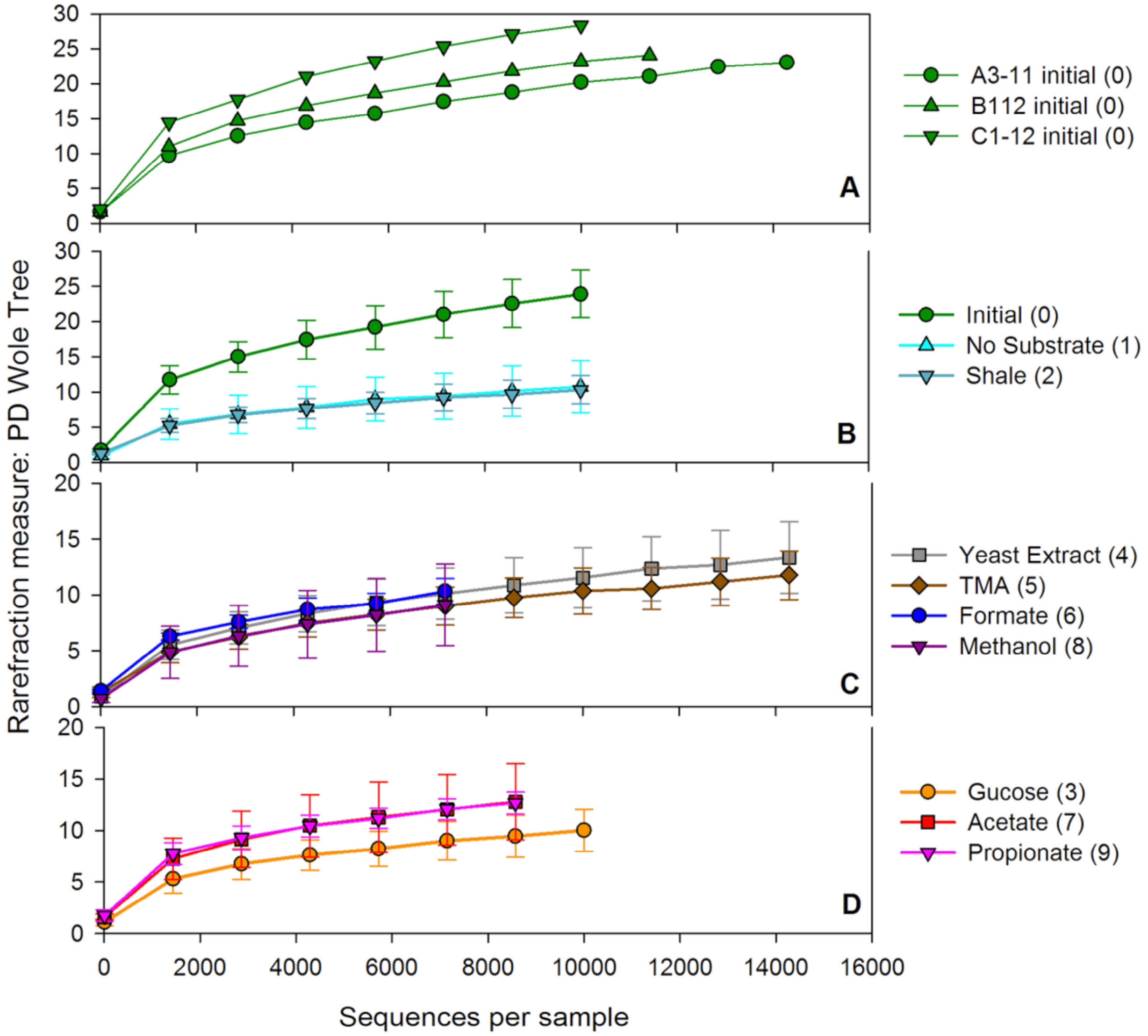

Supplementary Fig. S2

Comparison of rarefraction α diversity measures in the initial well waters and upon completion of the various substrate incubation experiments including the no-substrate control using the Phylogenetic Diversity metric. Grouping of treatments is based on (A) initial well waters (B) Natural or no substrates (C) substrates that resulted in methane production (D) substrates that did not yield methane.

	Treatment	Seqs/Sample	PD Ave.	PD Err.	chao1 Ave.	chao1 Err.	obs. Spec. Ave.	obs. Spec. Err.
0	Initial	10	1.757	0.213	9.133	2.331	5.233	0.772
	Initial	1437	11.726	1.993	220.458	39.248	84.733	14.474
	Initial	2864	14.989	2.143	329.281	46.159	130.167	22.431
	Initial	4291	17.427	2.709	377.636	65.91	164.267	27.897
	Initial	5718	19.189	3.086	416.517	54.082	193.133	33.98
	Initial	7145	21.003	3.27	451.916	72.936	221.3	38.618
	Initial	8572	22.542	3.414	508.594	60.058	247.567	41.488
	Initial	9999	23.897	3.372	563.688	40.041	271.567	42.962
1	No Substrate	10	0.968	0.422	5.1	3.645	3.167	1.372
	No Substrate	1437	5.47	2.127	112.941	33.97	42.867	11.493
	No Substrate	2864	6.828	2.728	161.493	59.006	65.8	17.759
	No Substrate	4291	7.82	2.942	204.445	53.311	83.033	22.09
	No Substrate	5718	8.986	3.055	261.285	86.889	101.167	25.601
	No Substrate	7145	9.404	3.257	263.178	81.236	112.133	28.74
	No Substrate	8572	10.145	3.589	280.728	103.957	125.033	34.065
	No Substrate	9999	10.763	3.707	293.635	102.989	138.033	37.105
2	Shale	10	1.3	0.156	3.733	0.484	3.133	0.33
	Shale	1437	5.231	0.965	95.954	44.029	37.433	9.468
	Shale	2864	6.721	1.106	145.272	48.495	56.4	12.942
	Shale	4291	7.646	1.454	160.783	58.607	71.133	17.466
	Shale	5718	8.428	1.529	184.978	62.456	83.867	20.956
	Shale	7145	9.219	1.896	221.259	79.966	97.533	28.152
	Shale	8572	9.658	1.993	241.998	94.606	107.533	32.008
	Shale	9999	10.298	1.993	263.654	91.972	118.933	33.595
3	Glucose	10	1.088	0.331	3.139	1.446	2.767	0.998
	Glucose	1437	5.296	1.4	82.775	26.924	34.833	11.238
	Glucose	2864	6.759	1.498	139.338	29.399	53.3	15.423
	Glucose	4291	7.622	1.469	176.719	47.704	67.233	19.566
	Glucose	5718	8.224	1.706	185.939	42.022	79.633	21.273
	Glucose	7145	8.988	1.862	211.594	70.179	89.767	25.485
	Glucose	8572	9.448	2.019	223.835	62.795	99.333	28.686
	Glucose	9999	10.014	2.033	243.836	48.884	108.867	29.604
4	Yeast Extract	10	0.928	0.539	3.383	0.968	2.567	0.464
	Yeast Extract	1437	5.522	1.309	127.231	18.056	51.533	8.328
	Yeast Extract	2864	7.041	1.434	164.595	5.739	78.067	12.597
	Yeast Extract	4291	8.338	1.615	191.128	18.457	98.933	11.726
	Yeast Extract	5718	9.322	2.07	228.007	20.871	113.8	13.425
	Yeast Extract	7145	10.115	2.27	240.224	24.024	128.033	12.283
	Yeast Extract	8572	10.857	2.476	254.004	15.598	139.533	11.005
	Yeast Extract	9999	11.542	2.682	288.275	29.896	151.267	10.844
	Yeast Extract	11426	12.35	2.864	307.393	34.974	164.733	8.693
	Yeast Extract	12853	12.676	3.073	323.78	38.118	171.267	8.157
	Yeast Extract	14280	13.338	3.182	328.283	34.614	179.667	8.122

	Treatment	Seqs/Sample	PD Ave.	PD Err.	chao1 Ave.	chao1 Err.	obs. Spec. Ave.	obs. Spec. Err.
5	TMA	10	1.313	0.458	4.917	3.317	3.65	2.05
	TMA	1437	4.912	0.988	104.871	50.382	46.55	18.95
	TMA	2864	6.251	1.106	184.69	67.791	72.3	27.4
	TMA	4291	7.477	1.234	208.523	71.463	93.5	34.6
	TMA	5718	8.266	1.38	255.776	91.699	108.9	42.1
	TMA	7145	9.019	1.703	243.579	85.871	124.75	47.15
	TMA	8572	9.749	1.764	275.461	105.633	137.35	52.15
	TMA	9999	10.354	2.058	297.704	115.056	151.25	56.05
	TMA	11426	10.575	1.862	314.638	107.858	163.95	59.45
	TMA	12853	11.162	2.117	345.699	148.502	175.1	65.5
	TMA	14280	11.773	2.184	325.967	132.227	185.75	68.25
6	Formate	10	1.437	0.124	4.25	0.442	3.333	0.419
	Formate	1437	6.293	0.299	100.837	15.98	43.7	3.78
	Formate	2864	7.594	0.593	140.171	9.354	63.1	4.704
	Formate	4291	8.706	1.017	194.492	43.249	79.4	9.504
	Formate	5718	9.239	0.897	194.847	31.88	91.8	9.476
	Formate	7145	10.322	1.159	238.567	50.121	105.967	11.625
7	Acetate	10	1.595	0.292	7.35	2.48	4.567	1.239
	Acetate	1437	7.253	2.029	132.608	53.177	53.133	15.598
	Acetate	2864	9.129	2.713	193.125	74.803	80.867	25.217
	Acetate	4291	10.453	3.033	229.247	76.325	101.467	31.628
	Acetate	5718	11.27	3.423	253.907	73.332	120.4	39.538
	Acetate	7145	12.023	3.419	308.948	86.918	134.8	43.093
	Acetate	8572	12.786	3.69	344.976	102.923	150.367	48.853
8	Methanol	10	0.749	0.38	3.183	1.532	2.333	0.685
	Methanol	1437	4.864	2.334	104.011	38.101	45.067	15.99
	Methanol	2864	6.336	2.721	140.757	24.73	65.133	24.08
	Methanol	4291	7.384	3.026	183.258	42.469	82.767	28.84
	Methanol	5718	8.206	3.237	224.758	85.566	97.133	32.457
	Methanol	7145	9.109	3.625	246.05	109.04	110.6	36.944
9	Propionate	10	1.735	0.544	9.325	4.625	4.95	1.95
	Propionate	1437	7.752	1.061	142.601	18.939	59.4	12.5
	Propionate	2864	9.284	1.128	196.292	22.527	86.95	16.65
	Propionate	4291	10.422	1.045	261.299	25.025	112.4	17
	Propionate	5718	11.16	0.992	289.656	20.569	130.4	19.1
	Propionate	7145	12.063	1.039	338.871	23.439	148.2	22
	Propionate	8572	12.665	1.07	380.485	21.779	166.85	21.05

Supplementary Table S3. Comparison of rarefraction α diversity measures in the initial well waters and upon completion of the various substrate incubation experiments including the no-substrate control using the following metrics: Phylogenetic Diversity (PD whole tree; a measure showing the branch length on a phylogenetic tree is covered by a given sample); Number of estimated species using the Chao1 estimator of species richness; and number of observed species-level OTUs (at the 97% level) in each community. Average values for each substrate and standard deviation are shown. A graphical presentation for the rarefraction curves of initial well waters vs. treatments using the phylogenetic diversity metrics is shown in Supplementary Fig. S2.

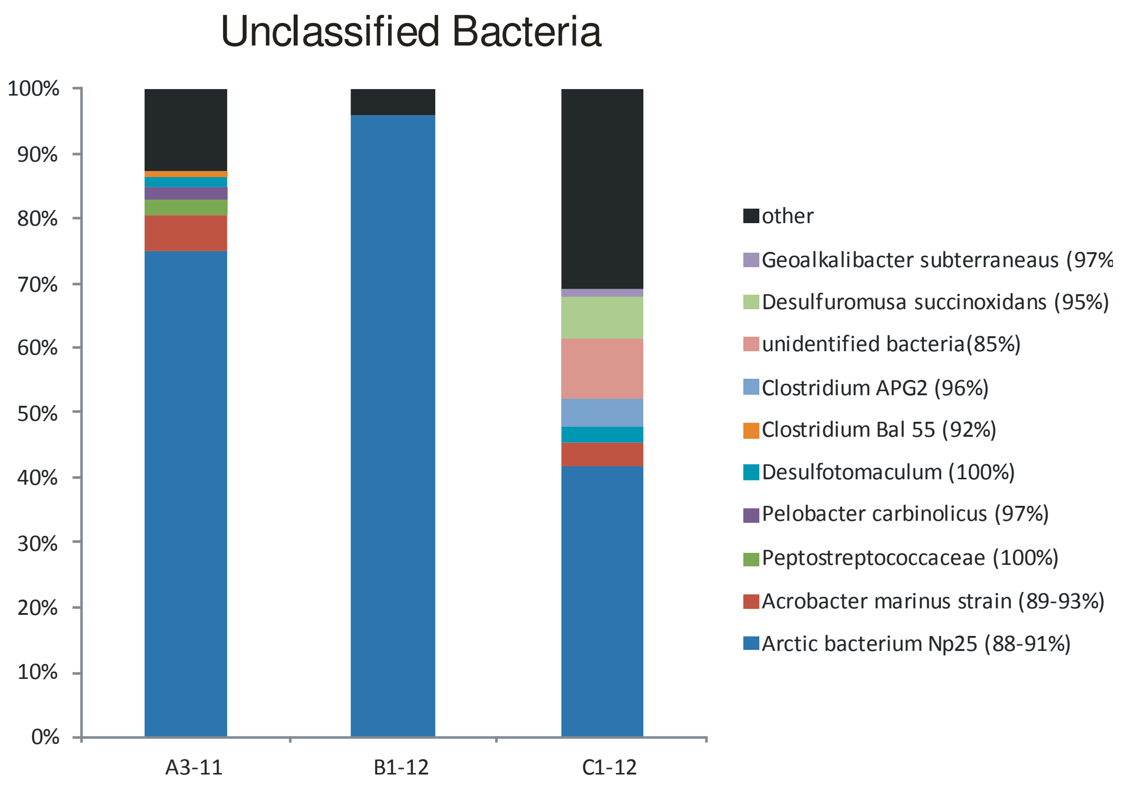

Supplementary Fig. S3: Most similar sequences from RDP to OTUs within the "unclassified bacteria group", and which represented >1% of the total Illumina reads in the unclassified bacteria cluster. "Other" shows sum of OTUs which represent less than 1% of the total Illumina reads in the unclassified bacteria cluster.

Supplementary Table S4. Comparison between bacterial OTUs in well waters identified from capillary sequencing of DGGE bands and Illumina MiSeq analysis

NCBI accession nr	Illumina OTU ID	closets culture NCBI hit	identity %	closest environmental NCBI hit	identity %
	**Firmicutes:**				
KF728891	1742	Halanaerobium hydrogeniformans	99	this study DGGE_ OTU1_1	99
KF728892	437	Halanaerobium congolense	100	this study DGGE_OTU1_2	100
KF728893	265	Orenia salinaria	90	this study DGGE_OTU14	99
KF728894	615	Orenia marismotui	94	this study DGGE_OTU13	100
KF728895	1072	Halocella cellulolsilytica	96	this study DGGE_OTU2	99
	**Bacteroidetes**				
KF728896	1443	Cytophaga sp. AN-BI4	100	this study DGGE_ OTU35	100
KF728897	1364	Marinilabilia salmonicolor	100	this study DGGE_OTU34	100
KF728898	138	Prolixibacter bellariivorans	95	this study DGGE_OTU37	100
KF728899	975	Bacteroidetes bacterium G13a-B	100	this study DGGE_OTU38	100
KF728900	805	Bacterium Phenol-4	98	uncultured bacterium	100
KF728901	667	Bacteroides graminisolvens	100	uncultured bacterium	100
	**Proteobacteria**				
KF728902	148	Arcobacter marinus	100	this study DGGE_OTU25	100
KF728903	1339	Sulfurospirillum carboxydovorans	99	this study DGGE_ OTU27	99
KF728904	1363	Pelobacter carbinolicus	100	this study DGGE_OTU18/20	100
KF728905	665	Desulfovibrio sp. AND1	99	this study DGGE_OTU16	99
KF728906	99	Geobacter hephaestius	98	Uncultured Geobacter	99
KF728907	1407	Sulfurospirillum halorespirans	92	this study DGGE_OTU26	99
KF728908	135	Geothermobacter sp. HR-1	98	Uncultured Geobacter	98
KF728909	1362	Desulfuromusa	98	this study DGGE_ OTU20	100
KF728910	323	Geoalkalibacter subterraneus	100	this study DGGE_OTU21	100
KF728911	464	Desulfovibrio aespoeensis Aspo-2	100	this studu DGGE_OTU15	100
	**unclassified Bacteria**				
KF728912	772	Arctic bacterium NP25	91	this study DGGE_OTU28	100
KF728913	388	Acrobacter marinus	93	this study DGGE_OTU25	93
KF728914	67	Peptostreptococcaceae Col 18	99	this study DGGE_OTU 9	99
KF728915	590	Pelobacter carbinolicus	97	this study DGGE_OTU 18	97
KF728916	66	Desulfotomaculum halophilum	100	Desulfotomaculum halophilum	100
KF728917	452	Clostridium Bal 55	92	this study DGGE_OTU11	99
KF728918	1243	Acrobacter marinus	89	this study DGGE_OTU25	89
KF728922	1123	unidentifyed bacteria from soil	85	GB vent bacterium	96
KF728919	840	Desulfuromusa succinoxidans	95	this study DGGE_OTU 20	97
KF728920	446	Clostridium APG2	96	this study DGGE_ OTU 8	100
KF728921	310	Geoalkalibacter subterraneaus	97	this study DGGE_OTU21	97
	**Archaea**				
KF728923	430	Methanohalophilus halophilus	99	this study DGGE OTU_M1	100
KF728924	869	Methanolobus profundi	99	this study DGGE OTU_M1_9	100
KF728925	124	Methanoplanus limicola	99	Uncultured archaeon clone MIARCCh10	99
KF728925	124	Methanoplanus limicola	99	this study DGGE OTU_M2	94
KF728926	481	Methanocalculus halotolerans	98	this study DGGE OTU_M3	98
KF728927	84	Euryarchaeote D4.75-18	90	Uncultured euryarchaeote clone ANT144-BP	97
KF728927	84	Euryarchaeote D4.75-18	90	this study DGGE OTU_M1_9	96
KF728928	1797	Euryarchaeote D4.75-18	88	Uncultured archaeon clone O127706F11 (2)	94
KF728929	1456	Euryarchaeote D4.75-18	91	Uncultured euryarchaeote clone GN27N3B (3)	94
KF728930	1434	Euryarchaeote D4.75-18	87	Uncultured archaeon DGGE gel band 15 (5)	94
KF728931	1608	Euryarchaeote D4.75-18	88	Uncultured euryarchaeote clone ANT106-CF (7)	99
KF728932	1839	Euryarchaeote D4.75-18	87	Uncultured euryarchaeote clone TD01 (8)	92
KF728933	1574	Euryarchaeote D4.75-18	90	Uncultured archaeal clone ANNA-A8 (11)	94
KF728934	872	Archaeoglobus infectus	90	Uncultured archaeon clone MIARCBf08 (1)	98
KF728935	1429	Archaeoglobus infectus	90	Uncultured archaeon clone MIARCBf08 (1)	100
KF728936	475	Archaeoglobus infectus	90	Uncultured archaeon clone MIARCBf08 (1)	97
KF728937	417	Euryarchaeote J3.25-8	91	Uncultured archaeon clone CP-A123	99
KF728937	417	Euryarchaeote J3.25-8	91	this study DGGE OTU_O5	97
KF728938	1829	no close match		this study OTU_D1_9	98

KF728939	919	no close match	this study OTU_D1_9	96
KF728940	831	no close match	this study OUT_D1_9	94
KF728941	130	no close match	Uncultured euryarchaeote clone ANT144-BP	97
KF728941	130	no close match	this study DGGE OTU_M1_9	97
KF728942	976	no close match	Uncultured archaeon clone KSTwh-C1-1-A-033 (6)	96
KF728943	855	no close match	Uncultured archaeon OUT_4a (10)	83
KF728944	586	no close match	Uncultured euryarchaeote clone GN25D01A (9)	92
KF728945	1563	no close match	Uncultured archaeon clone KSTwh-C1-1-A-033 (6)	92
KF728946	578	no close match	Uncultured euryarchaeote clone GN28DB56 (4)	89

Supplementary Table S5: All Bacterial (A) and Archaeal (B) OTUs with accession numbers recovered with the DGGE approach in the initial formation water and incubation experiments. OTUs marked with * were only found in yeast extract and TMA incubation experiments but not in the initial well water. Maximal identities to closest cultured relatives from NCBI with their accession number are shown.

This study OTUnr	This study NCBI Accession nr	Max ident	closest cutured relative NCBI Description	closest cutured relative NCBI Accession nr
Bacteria:				
1_1	KC262274	100%	*Halanaerobium hydrogeniformans*	CP002304
1_2	KC262275	99%	*Halanaerobium congolense* strain SEBR 4224	NR_026044
2	KC262276	93%	*Halocella cellulolsilytica*	NR_036959
3	KC262277	96%	*Sporosalibacterium faouarense*	EU567322
4	KC262278	98%	*Sporosalibacterium faouarense*	EU567322
5	KC262279	96%	*Clostridium caminithermale* strain DVird3	NR_041887
6	KC262280	99%	*Clostridium* sp. AN-AS3B	FR872929
7*	KC262281	96%	*Alkaliphilus peptidofermentans* strain Z-7036	EF382660
8	KC262282	95%	*Clostridium* sp. AN-AS6C	FR872932
9	KC262283	100%	Peptostreptococcaceae bacterium Col 18	GU194175
10	KC262284	97%	*Clostridium caminithermale* strain DVird3	NR_041887
11	KC262285	92%	Clostridiales bacterium Bal55	AB260037
12	KC262286	93%	Clostridiales bacterium Bal55	AB260037
13	KC262287	92%	*Orenia marismortui* strain DSM 5156	NR_026259
14	KC262288	94%	*Orenia marismortui* strain DSM 5156	NR_026259
15	KC262289	99%	*Desulfovibrio aespoeensis* Aspo-2	CP00243
16	KC262290	100%	*Desulfovibrio* sp. AND1	AY281344
17	KC262291	99%	*Desulfovibrio longus* strain SEBR 2582	NR_025765
18	KC262292	99%	*Pelobacter venetianus*	NR_044779
20	KC262293	92%	*Pelobacter carbinolicus*	U23141
21	KC262294	99%	*Geoalkalibacter subterraneus* strain Red1	NR_044429
22	KC262295	100%	e.g. *Pseudomonas salomonii* strain +Y14	JX134631
23	KC262296	100%	e.g. *Pseudomonas sp.* A15 (2012)	JQ522968
25	KC262298	100%	*Arcobacter marinus* strain CL-S1	EU512920
26	KC262299	93%	*Sulfurospirillum* sp. 18.1	AF357199
27	KC262300	99%	*Geospirillum* sp. SM-5	U85965
27	KC262300	99%	*Sulfurospirillum carboxydovorans* strain MV	AY740528
28	KC262301	96%	Arctic bacterium NP25	EU196331
29	KC262302	96%	Arctic bacterium NP25	EU196331
30	KC262303	100%	*Alcaligenes* sp. ES-JQ-2	FJ529027
31	KC262304	97%	*Dechloromonas* UWNR4	FJ477303
32*	KC262305	100%	*Dethiosulfovibrio acidaminovorans*	NR_029034
32*	KC262305	100%	*Dethiosulfovibrio russensis*	NR_041793
32*	KC262305	100%	*Dethiosulfovibrio marinus*	NR_025081
33	KC262306	89%	*Aminobacterium colombiense* DSM 12261	CP001997
34	KC262307	99%	*Marinilabilia salmonicolor*	GU198996
35	KC262308	98%	*Cytophaga* sp. AN-BI4	AM157648
36	KC262309	99%	*Prolixibacter bellariivorans* strain F2	NR_043273
37	KC262310	96%	*Prolixibacter bellariivorans* strain F2	NR_043273
38	KC262311	98%	Bacteroidetes bacterium G13a-B	FN397996
40	KC262312	100%	Propionibacterium sp. S4-S6	JX104050
41	KC262313	100%	Corynebacterium sp. VTT E-073034	EU438939
42	KC262314	94%	*Alkalibaculum bacchi* strain CP11	FJ438469

(A)

This study OTUnr		This study NCBI Accession nr	Max ident	closest cutured relative NCBI Description	closest cutured relative NCBI Accession nr
Archaea:					
M1_59		KC262315	99-100%	*Methanolobus / Methanohalophilus*	NR_041665/JQ346755
M1_9		KC262316	99-100%	*Methanolobus / Methanohalophilus*	NR_041665/JQ346756
M1_45		KC262317	99-100%	*Methanolobus / Methanohalophilus*	NR_041665/JQ346757
M1_0426a003		KC262318	99-100%	*Methanolobus / Methanohalophilus*	NR_041665/JQ346758
M1_0426a002		KC262319	99-100%	*Methanolobus / Methanohalophilus*	NR_041665/JQ346759
M1_0426a063		KC262320	99-100%	*Methanolobus / Methanohalophilus*	NR_041665/JQ346760
M1_0425a007		KC262321	99-100%	*Methanolobus / Methanohalophilus*	NR_041665/JQ346761
M1_0330a064		KC262322	99-100%	*Methanolobus / Methanohalophilus*	NR_041665/JQ346762
M1_0406a002		KC262323	99-100%	*Methanolobus / Methanohalophilus*	NR_041665/JQ346763
M1_0406a010		KC262324	99-100%	*Methanolobus / Methanohalophilus*	NR_041665/JQ346764
M1_0406a056		KC262325	99-100%	*Methanolobus / Methanohalophilus*	NR_041665/JQ346765
M2_0406a013		KC262326	99%	*Methanoplanus limicola*	AB546259
M3_0427a021		KC262327	100%	*Methanocalculus halotolerans*	NR_024870
M3_0427a017		KC262328	100%	*Methanocalculus halotolerans*	NR_024870
				closest environmental sequence NCBI	
D1_9	+	KC262329	94%	uncultured archaeon clone 1572_93arch	JF500394
D1_1201a019	+	KC262330	96%	uncultured euryachaeote clone ANT-144BP	GU969471
O1_5	+	KC262331	86%	uncultured archaeon clone SS030	EU329767
O2_5	+	KC262332	84%	uncultured archaeon clone SS030	EU329767
O3_2	+	KC262333	96%	uncultured archaeon clone CP-A 127	DQ521194
O4_1128a090	+	KC262334	83%	uncultured archaeon clone ss036a	AJ969774
O5_3		KC262335	95%	uncultured archaeon clone GH-A 55	DG521145

(**B**)

Archaeal OTUs marked with + were only found in the initial well water but not in the incubation experiments.

Supplementary Fig. S4 (next page): Maximum likelihood tree of euryarchaeal 16S rRNA genes built using RAxML on 1323 aligned positions, showing the phylogenetic relationship between DGGE fragments detected in this study and major euryarchaeal groups. Accession numbers are shown for all sequences obtained from Genbank. Nodes are labeled with the percentage of bootstrap variations (out of 100) that contained that node. Unlabeled nodes denote positions at which short or low-quality sequences were added to the tree using ARB's parsimony quick-add functionality, and the species are shown in bold. The number of species included in collapsed groups is shown to the right of their position. Sequences marked with an * represent previously described OTUs from the Antrim Shale environment (Waldron *et al.*, 2007). Sequences from this study are color coded in grey.

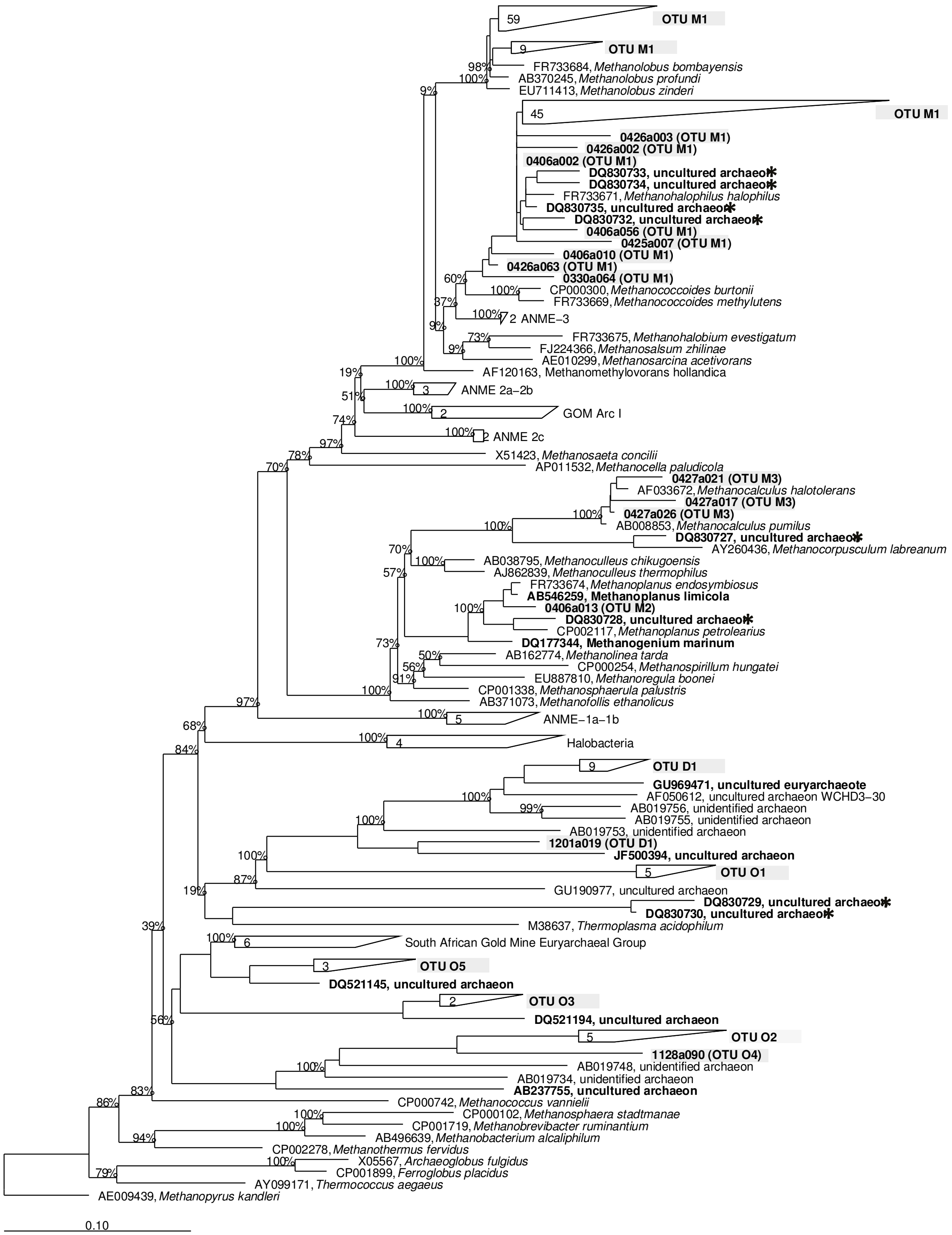
OTU M1
OTU M1
FR733684, Methanolobus bombayensis
AB370245, Methanolobus profundi
EU711413, Methanolobus zinderi
OTU M1
0426a003 (OTU M1)
0426a002 (OTU M1)
0406a002 (OTU M1)
DQ830733, uncultured archaeon
DQ830734, uncultured archaeon
FR733671, Methanohalophilus halophilus
DQ830735, uncultured archaeon
DQ830732, uncultured archaeon
0406a056 (OTU M1)
0425a007 (OTU M1)
0406a010 (OTU M1)
0426a063 (OTU M1)
0330a064 (OTU M1)
CP000300, Methanococcoides burtonii
FR733669, Methanococcoides methylutens
ANME-3
FR733675, Methanohalobium evestigatum
FJ224366, Methanosalsum zhilinae
AE010299, Methanosarcina acetivorans
AF120163, Methanomethylovorans hollandica
ANME 2a-2b
GOM Arc I
ANME 2c
X51423, Methanosaeta concilii
AP011532, Methanocella paludicola
0427a021 (OTU M3)
AF033672, Methanocalculus halotolerans
0427a017 (OTU M3)
0427a026 (OTU M3)
AB008853, Methanocalculus pumilus
DQ830727, uncultured archaeon
AY260436, Methanocorpusculum labreanum
AB038795, Methanoculleus chikugoensis
AJ862839, Methanoculleus thermophilus
FR733674, Methanoplanus endosymbiosus
AB546259, Methanoplanus limicola
0406a013 (OTU M2)
DQ830728, uncultured archaeon
CP002117, Methanoplanus petrolearius
DQ177344, Methanogenium marinum
AB162774, Methanolinea tarda
CP000254, Methanospirillum hungatei
EU887810, Methanoregula boonei
CP001338, Methanosphaerula palustris
AB371073, Methanofollis ethanolicus
ANME-1a-1b
Halobacteria
OTU D1
GU969471, uncultured euryarchaeote
AF050612, uncultured archaeon WCHD3-30
AB019756, unidentified archaeon
AB019755, unidentified archaeon
AB019753, unidentified archaeon
1201a019 (OTU D1)
JF500394, uncultured archaeon
OTU O1
GU190977, uncultured archaeon
DQ830729, uncultured archaeon
DQ830730, uncultured archaeon
M38637, Thermoplasma acidophilum
South African Gold Mine Euryarchaeal Group
OTU O5
DQ521145, uncultured archaeon
OTU O3
DQ521194, uncultured archaeon
OTU O2
1128a090 (OTU O4)
AB019748, unidentified archaeon
AB019734, unidentified archaeon
AB237755, uncultured archaeon
CP000742, Methanococcus vannielii
CP000102, Methanosphaera stadtmanae
CP001719, Methanobrevibacter ruminantium
AB496639, Methanobacterium alcaliphilum
CP002278, Methanothermus fervidus
X05567, Archaeoglobus fulgidus
CP001899, Ferroglobus placidus
AY099171, Thermococcus aegaeus
AE009439, Methanopyrus kandleri
0.10

Fig. S4

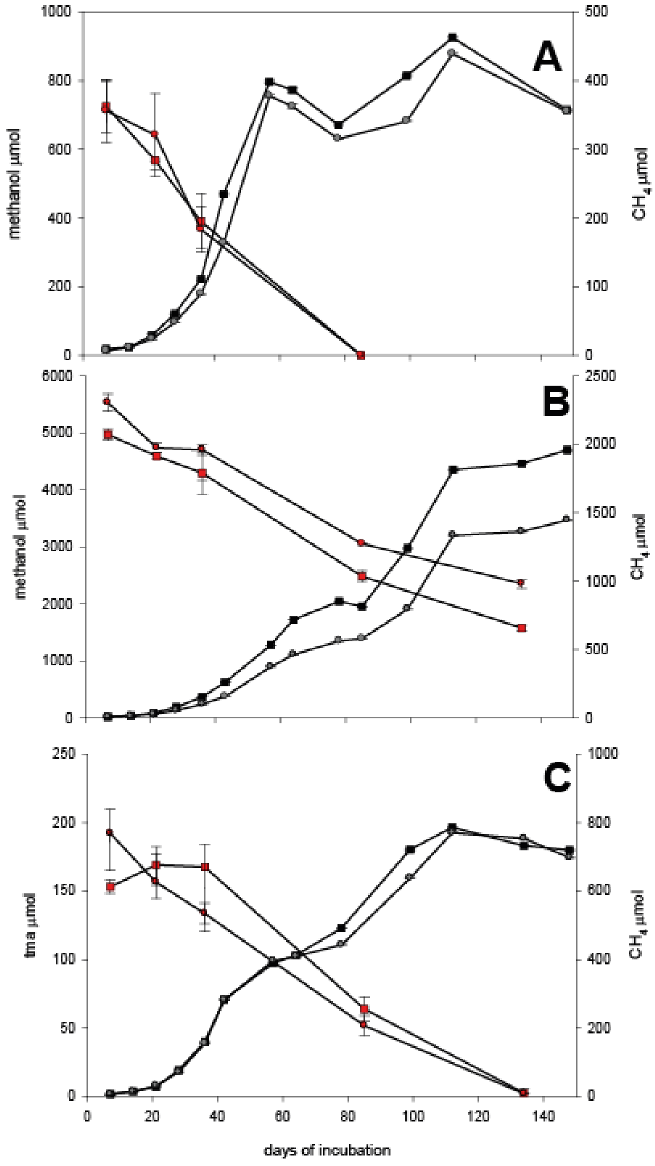

Supplementary Fig. S5: Methanol and TMA consumption, and methane accumulation in the A3-11 well over the course of the experiment. (A) no substrate (B) methanol addition (C) TMA addition.

Methane yields: ■ incubation bottle 1, ● incubation bottle 2.

Methanol and TMA amounts: ■ incubation bottle 1, ● incubation bottle 2.

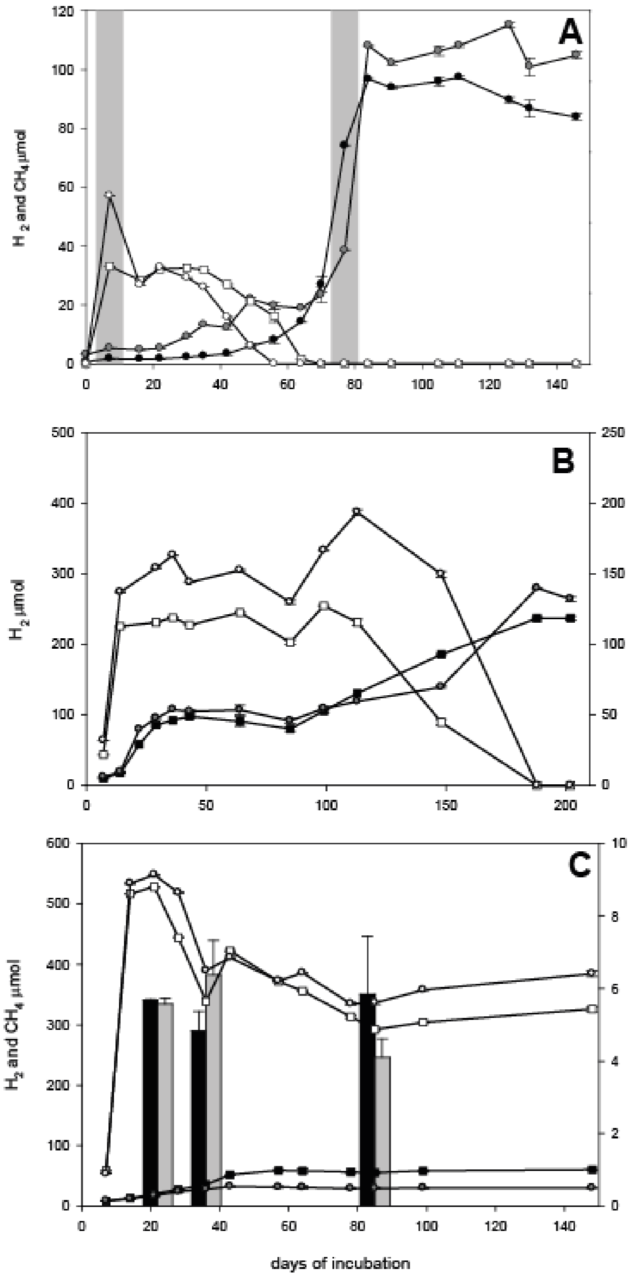

Supplementary Fig. S6: Hydrogen and methane accumulation in different treatments: (A) yeast extract C1-12 well, shaded gray area marks sampling for amino acid analyses, (B) formate B1-12 well and (C) glucose A3-11 well.

Methane accumulation: ■ incubation bottle 1, ● incubation bottle 2,

Hydrogen accumulation: □ incubation bottle 1, ○ incubation bottle 2,

Ethanol amounts: ▬ incubation bottle 1, ▭ incubation bottle 2.

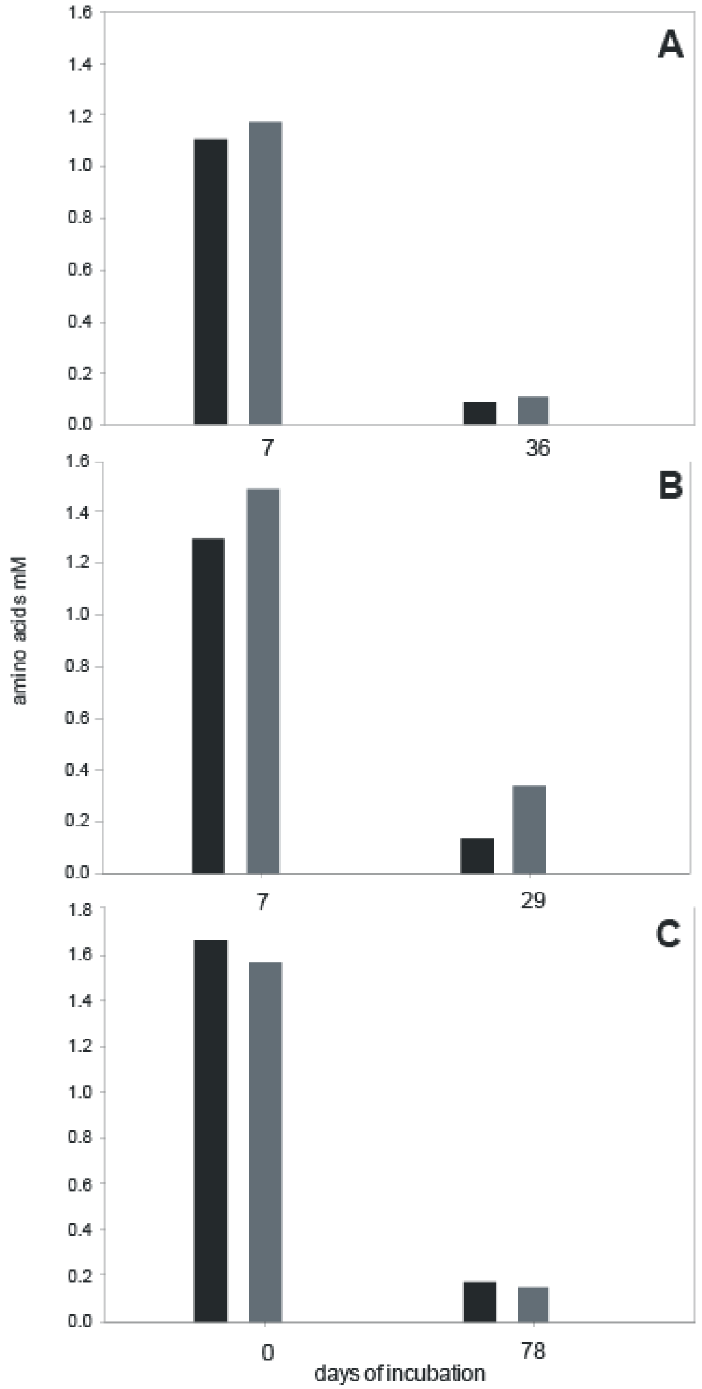

Supplementary Fig. S7: Amino acid consumption in the yeast extract incubation. **A**: A3-11 well, **B**: B1-12 well, **C**: C1-12 well. Black: bottle 1, dark grey: bottle 2.

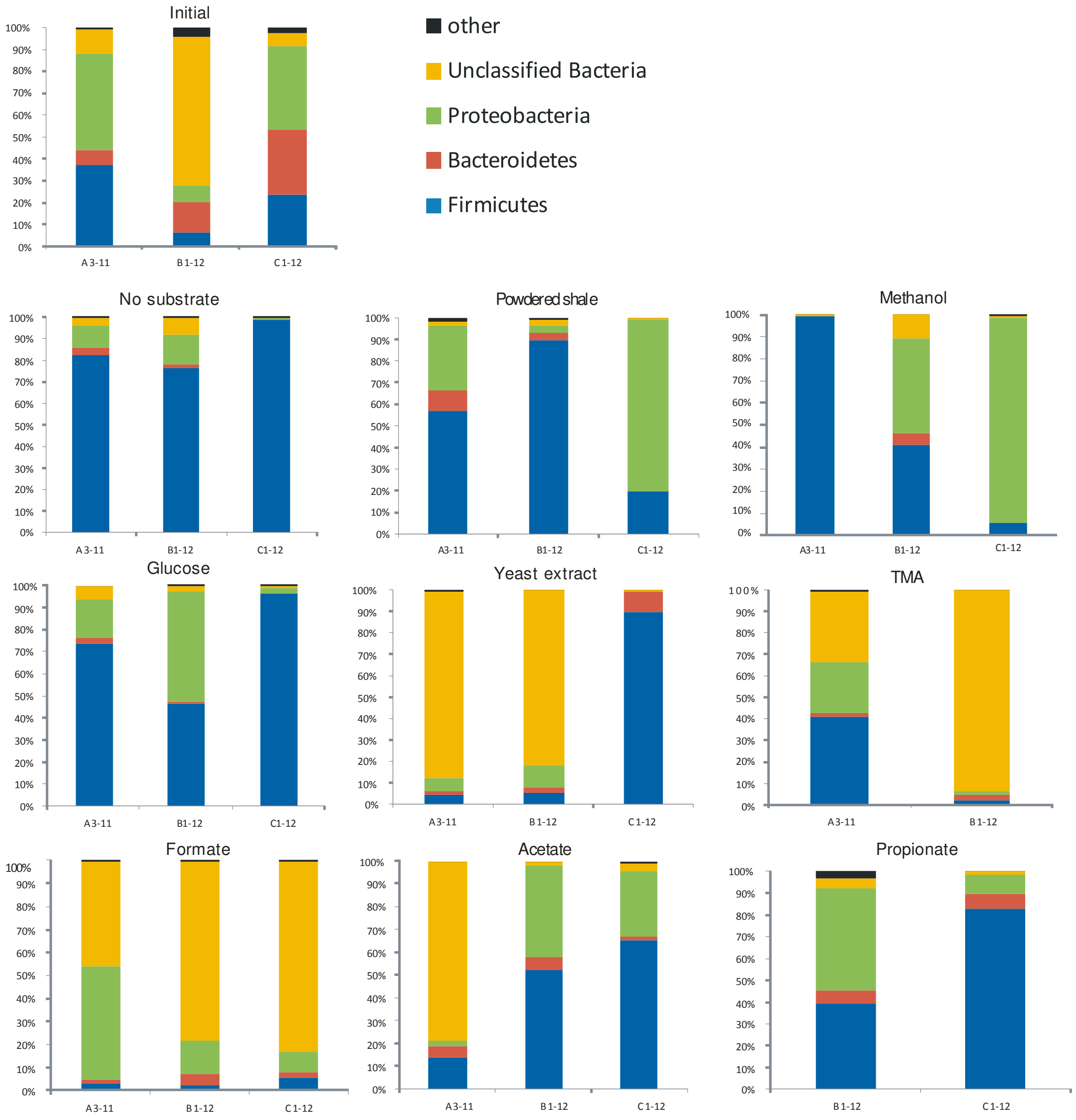

Supplementary Fig. S8: Detailed information of the prokaryotic community composition in the different incubation experiments. Equal amounts of PCR products from the duplicate bottles were pooled for subsequent Illumina sequencing.

Bacterial reads represent >97% of the total Illumina reads. The dominant OTUs in the different phyla were closest related to cultures from:

- Firmicutes: *Halanerobium hydrogenoformans*, *-congolense* and *Orenia marismortui*,-*salinaria*,
- Bacteroidetes: *Marinilabilia salmonicolor*, *Prolixibacter bellariivorans*.
- Proteobacteria: *Acrobacter marinus*, *Pelobacter carbinolicus*, *Sulfurospirillum* sp. and *Desulfovibrio* sp.
- Unclassified bacteria: Arctic bacterium NP25

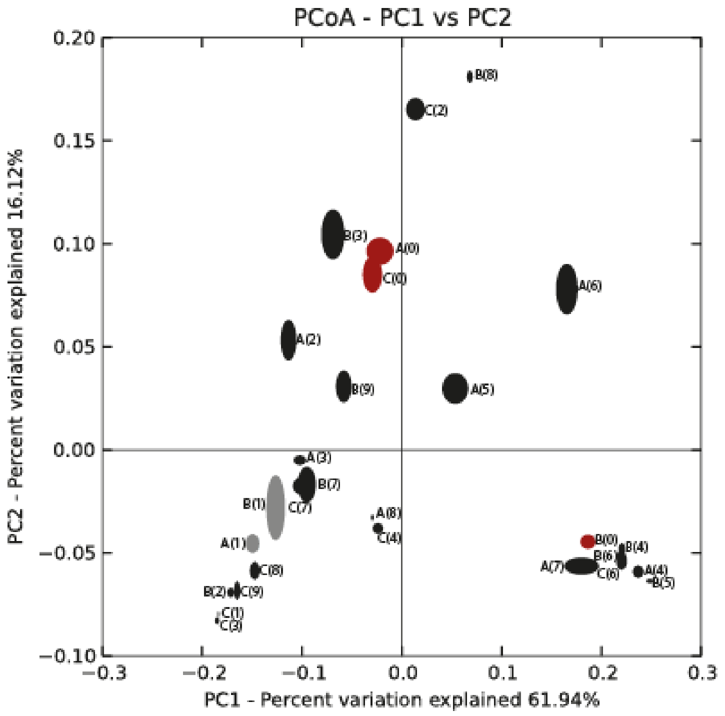

Supplementary Fig. S9. Jackknifed PCoA plot of initial well water samples and after incubation with the various methanogenic and fermentative substrates with weighted Unifrac. Shown is a plot of the first two principal coordinate axes, which combined explain 78% of the variation. Ellipses represent the interquartile range of the distribution of points among the ten jackknifed replicates. A (A3-11), B (B1-12), C (1-12), 0 (red: initial well water), 1 (grey: no substrate incubation control), 2 (shale), 3 (glucose), 4 (yeast extract), 5 (TMA), 6 (formate), 7 (acetate), 8 (methanol), 9 (propionate).

References

Altschul SF, Gish W, Miller W, Myers EW & Lipman DJ (1990) Basic Local Alignment Search Tool. *Journal of Molecular Biology* **215**: 403-410.

Coolen MJL, Saenz JP, Giosan L, Trowbridge NY, Dimitrov P, Dimitrov D & Eglinton TI (2009) DNA and lipid molecular stratigraphic records of haptophyte succession in the Black Sea during the Holocene. *Earth and Planetary Science Letters* **284**: 610-621.

DeLong EF (1992) Archaea in coastal marine environments. *Proceedings of the National Academy of Sciences of the United States of America* **89**: 5685-5689.

Ludwig W, Strunk O, Westram R*, et al.* (2004) ARB: a software environment for sequence data. *Nucleic Acids Research* **32**: 1363-1371.

Muyzer G, De Waal EC & Uitterlinden AG (1993) Profiling of complex microbial populations by denaturing gradient gel electrophoresis analysis of polymerase chain reaction-amplified genes coding for 16S rRNA. *Applied and Environmental Microbiology* **59**: 695-700.

Ovreas L, Forney L, Daae FL & Torsvik V (1997) Distribution of bacterioplankton in meromictic Lake Saelenvannet, as determined by denaturing gradient gel electrophoresis of PCR-amplified gene fragments coding for 16S rRNA. *Applied and Environmental Microbiology* **63**: 3367-3373.

Pruesse E, Quast C, Knittel K, Fuchs BM, Ludwig WG, Peplies J & Glockner FO (2007) SILVA: a comprehensive online resource for quality checked and aligned ribosomal RNA sequence data compatible with ARB. *Nucleic Acids Research* **35**: 7188-7196.

Schloss PD, Westcott SL, Ryabin T*, et al.* (2009) Introducing mothur: Open-Source, Platform-Independent, Community-Supported Software for Describing and Comparing Microbial Communities. *Applied and Environmental Microbiology* **75**: 7537-7541.

Stahl DA & Amann R (1991) Development and application of nucleic acid probes in bacterial systematics. *Nucleic acid techniques in bacterial systematics,*(Stackebrandt E & Goodfellow M, ed.^eds.), p.^pp. 205-248. Wiley, Chichester.

Takai K & Horikoshi K (2000) Rapid detection and quantification of members of the archaeal community by quantitative PCR using fluorogenic probes. *Applied and Environmental Microbiology* **66**: 5066-+.

Waldron PJ, Petsch ST, Martini AM & Nüsslein K (2007) Salinity constraints on subsurface archaeal diversity and methanogenesis in sedimentary rock rich in organic matter. *Applied and Environmental Microbiology* **73**: 4171-4179.

Wuchter C, Schouten S, Coolen MJL & Sinninghe Damste, JS (2004) Temperature-dependent variation in the distribution of tetraether membrane lipids of marine Crenarchaeota: Implications for TEX 86 paleothermometry. *Paleoceanography* **19**: PA4028, doi:4010.1029/2004PA001041, 002004-.
